# Iconicity in English and Spanish and Its Relation to Lexical Category and Age of Acquisition

**DOI:** 10.1371/journal.pone.0137147

**Published:** 2015-09-04

**Authors:** Lynn K. Perry, Marcus Perlman, Gary Lupyan

**Affiliations:** 1 University of Miami, Coral Gables, Florida, United States of America; 2 University of Wisconsin-Madison, Madison, Wisconsin, United States of America; Utrecht University, NETHERLANDS

## Abstract

Signed languages exhibit iconicity (resemblance between form and meaning) across their vocabulary, and many non-Indo-European spoken languages feature sizable classes of iconic words known as ideophones. In comparison, Indo-European languages like English and Spanish are believed to be arbitrary outside of a small number of onomatopoeic words. In three experiments with English and two with Spanish, we asked native speakers to rate the iconicity of ~600 words from the English and Spanish MacArthur-Bates Communicative Developmental Inventories. We found that iconicity in the words of both languages varied in a theoretically meaningful way with lexical category. In both languages, adjectives were rated as more iconic than nouns and function words, and corresponding to typological differences between English and Spanish in verb semantics, English verbs were rated as relatively iconic compared to Spanish verbs. We also found that both languages exhibited a negative relationship between iconicity ratings and age of acquisition. Words learned earlier tended to be more iconic, suggesting that iconicity in early vocabulary may aid word learning. Altogether these findings show that iconicity is a graded quality that pervades vocabularies of even the most “arbitrary” spoken languages. The findings provide compelling evidence that iconicity is an important property of all languages, signed and spoken, including Indo-European languages.

## Introduction

Nearly 2400 years ago, Plato contemplated whether the names of things are natural, or whether they are established by agreed upon convention [1]. His philosophical investigation illustrates the conflicted intuition of many scholars and laypeople alike: We often have the sense that words somehow sound like what they mean—that they are natural and correct—and yet, the forms of words with similar meanings often vary drastically within and between different languages. Contemporary linguistics and cognitive science have emphasized this latter point of *arbitrariness*, holding that outside of marginal cases of onomatopoeia (e.g., “buzz” and “bang”), languages are arbitrary by design [2]. As Pinker and Bloom observed, arbitrariness is “most obvious in the choice of individual words: there is no reason for you to call a dog *dog* rather than *cat* except for the fact that everyone else is doing it” (1990: p. 718).

However, in recent decades, research across the diverse languages of the world, both spoken and signed, has found considerable evidence that *iconicity*–a resemblance between form and meaning—is a widespread property of language [3–8]. In signed languages, many signs exhibit iconicity in the fairly direct and imitative resemblance between their form and meaning: for example, in British Sign Language (BSL), bringing a cupped hand close to the mouth to represent the act of “drinking.” Iconicity in signs can also take more abstract forms, as in the American Sign Language (ASL) classifier for long skinny objects, which is depicted with an upright index finger.

In contrast to signed languages, arbitrariness in spoken languages has often been taken as axiomatic, e.g., in the notion of Saussure’s “arbitrariness of the sign” [9]. Spoken words, on this view, have only trivial potential for iconicity owing to the limitations inherent to the auditory modality and thus spoken languages could simply be no other way (e.g., [10–12]). However, this strong claim for arbitrariness is called into question by a large number of studies showing evidence of iconicity in the words of spoken languages. (There is also a large literature showing that people share intuitions for the meanings expressed by various nonsense words and other sounds [13–17], These experiments demonstrate the potential for iconicity in vocalizations, but since they do not relate directly to iconicity existing in actual languages, we do not review them here in detail.)

One paradigm of experiments shows that people are better than chance at interpreting the meanings of antonyms in unfamiliar languages and unknown ethnozoological nomenclature in their own language, suggesting that they must be relying on iconic qualities of the words to do so [18–21]. For example, a classic study selected 21 pairs of English antonymic words referring to sense experiences (e.g. warm-cool, heavy-light), which were then translated into Chinese, Czech, and Hindi [18]. English-speaking participants unfamiliar with these languages were better than chance at correctly translating the antonym pairs back into their provided English words. Additionally, there is evidence from cross-linguistic studies that certain meanings tend to have similar forms across a representative sample of languages. For example, across languages, words that express diminutive meanings tend to contain high front vowels [22], and indexical words used to express close distance (e.g. “here”) tend to contain front vowels, whereas words expressing far distance (e.g. “there”) tend to have back vowels [23]. Another cross-linguistic study found that nasal consonants are common in words for ‘nose,’ whereas bilabial consonants are common in words for ‘mouth’ [24].

More broadly, linguists have documented rich inventories of iconic words in many languages, variously named expressives, mimetics—and most generally—ideophones [25–28]. These words, which are especially common outside of Indo-European languages can number in the thousands in a given language and tend to be grammatically and phonologically distinct from other word classes. For instance, in Japanese, the word ‘koron’ refers to a light object rolling once, ‘korokoro’ to a light object rolling repeatedly, and ‘gorogoro’ to a heavy object rolling repeatedly [3]. These instances illustrate the iconic use of reduplication to express repetition, and voiced (opposed to voiceless) consonants to express a more massive object. Across languages, ideophones are used to express a wide range of qualities like manner of movement and speed, luminance and color, shape, size, duration, texture, visual appearance, taste, temperature, and emotional and psychological states [26].

The body of cross-linguistic research showing iconicity in many of the words of spoken languages, especially the common existence of rich ideophone systems, indicates that iconicity is prevalent in the vocabularies of spoken languages too; it is not an exclusive property of signed languages. Recent accounts have even proposed that spoken languages that lack ideophone systems are actually the exception, rather than the rule [29]. According to this idea, the preoccupation with arbitrariness in linguistics and cognitive science arose from a focus on ideophone-poor Indo-European languages, especially English [29]. The refrain that there is no reason why a dog should be called a “dog” has obscured the possibility that iconicity may exist in these languages when one examines their vocabulary in a more methodical manner. Notably, iconicity is distinct from *systematicity*. Systematicity refers to language-internal statistical regularities between words and their meanings, which may be iconic or arbitrary [30,31]. For example, the sub-morphemic element “gl-”in English tends to relate to ‘light,’ giving a systematic clue to the meaning of words like “glimmer,” “glitter,” and “glow” [32]. Yet there is nothing obviously light-like about the “gl-”form; it is systematic but not necessarily iconic.

Here we asked native English and Spanish speakers to rate the iconicity of roughly 600 words from the MacArthur Bates Developmental Inventory (MCDI; [33]), a list of the words learned earliest by children (similar to the method used by [34,35]). We focused on English and Spanish because they are Indo-European languages thought to lack iconicity beyond fringe instances. Based on current theories of iconicity and its function in language [3,4,26], we hypothesized how iconicity would vary across the words of each language according to their *lexical category* and *age of acquisition*.

### Hypothesis 1: Iconicity and lexical category

Across languages, some types of words tend to be more iconic than other types. Perhaps the most paradigmatic case of iconic words are onomatopoeic words which use the sound of word to depict the sound of the referent. More generally, it has been suggested that adjectival and adverbial meanings, depicting aspects of sensory and motor experience are more likely to have iconic forms [3,26,36,37]. The expression of manner of movement and action is especially common, with ideophones most prevalent in languages that tend not to express this information within the verb ([23]; see verb-framed languages below). Ideophones are also used to express visual patterns and other sensory perceptions, and less frequently, inner feelings and cognitive states. Imai and Kita [3] note that while ideophones in Japanese and many other languages are rich in the expression of manners of actions, manners of physical sensations and certain properties of objects, few ideophones refer directly to objects. We therefore predicted that onomatopoeia and interjections (i.e., words derived from emotional vocalizations and scripted interactional routines) would be rated as most iconic in both English and Spanish. Adjectives would be rated as more iconic than nouns in both languages. Function words would be rated as least iconic in both languages owing to their more abstract meanings.

In hypothesizing about the iconicity of verbs, our predictions for English and Spanish diverge. English is a satellite-framed language. Information about the path of motion tends to be expressed in a prepositional phrase, as opposed to the verb [38]. Consequently English is free to incorporate information about the manner of motion directly into the semantics of the verb, which it frequently does. In contrast, Spanish is a verb-framed language, typically expressing the path of motion within the verb, and leaving manner to be optionally expressed by a prepositional phrase. For example, the English sentence “The bottle floated into the cave,” expresses the manner in which the bottle moved into the cave within the verb. In contrast, in Spanish the manner of movement is expressed separately from the verb [38]:

La botella entró a la cueva flotando

The bottle entered [into] the cave floating

Thus as English verbs are generally more expressive of manner of motion, we predicted that they would be rated as relatively high in iconicity, whereas Spanish verbs would be rated as relatively low.

### Hypothesis 2: Iconicity and age of acquisition

When children learn a spoken language, they face the difficult task of figuring out that vocalizations are ‘words’ with meanings that can serve as labels for various objects, actions, and properties. Language learning further demands that children extract the meanings of words from a noisy, complex environment, and properly generalize them to new exemplars. Considering these challenges, some researchers have proposed that iconicity could provide young learners with a valuable cue to constrain the potential meaning of a word [3,4]. In support of this hypothesis, experimental evidence shows that iconic words can be easier to learn for both children and adults [39–42]. For example, both Japanese- [41] and English-speaking toddlers [42] more accurately generalize novel iconic verbs than novel non-iconic verbs. Work with adults shows that when novel meanings are denoted by iconic words, the *meanings* themselves (the categories denoted by the words) are learned more quickly (26; also see 27). Research on learning of signed languages has found that, at least in British Sign Language, signs learned earlier in development were rated as more iconic by native signers [35,34]. We therefore predicted that the words of each language that are learned earlier in development would tend to be more iconic than words that are learned later.

## Experiment 1: English Words (written presentation)

We asked participants to rate the iconicity of 592 words from the MCDI, including 19 onomatopoetic words (10) and interjections (9), 60 adjectives, 99 verbs, 319 nouns, and 95 function words. Participants viewed the words in written form, and were instructed to say them aloud before making their rating (on a scale from -5 to 5 where -5 indicated a word sounded like the opposite of its meaning, 0 indicated a word was arbitrarily related to its meaning, and 5 indicated that a word was highly iconic). The instructions to the participants carefully defined iconicity and arbitrariness (see Methods).

### Results

**Fig 1:**
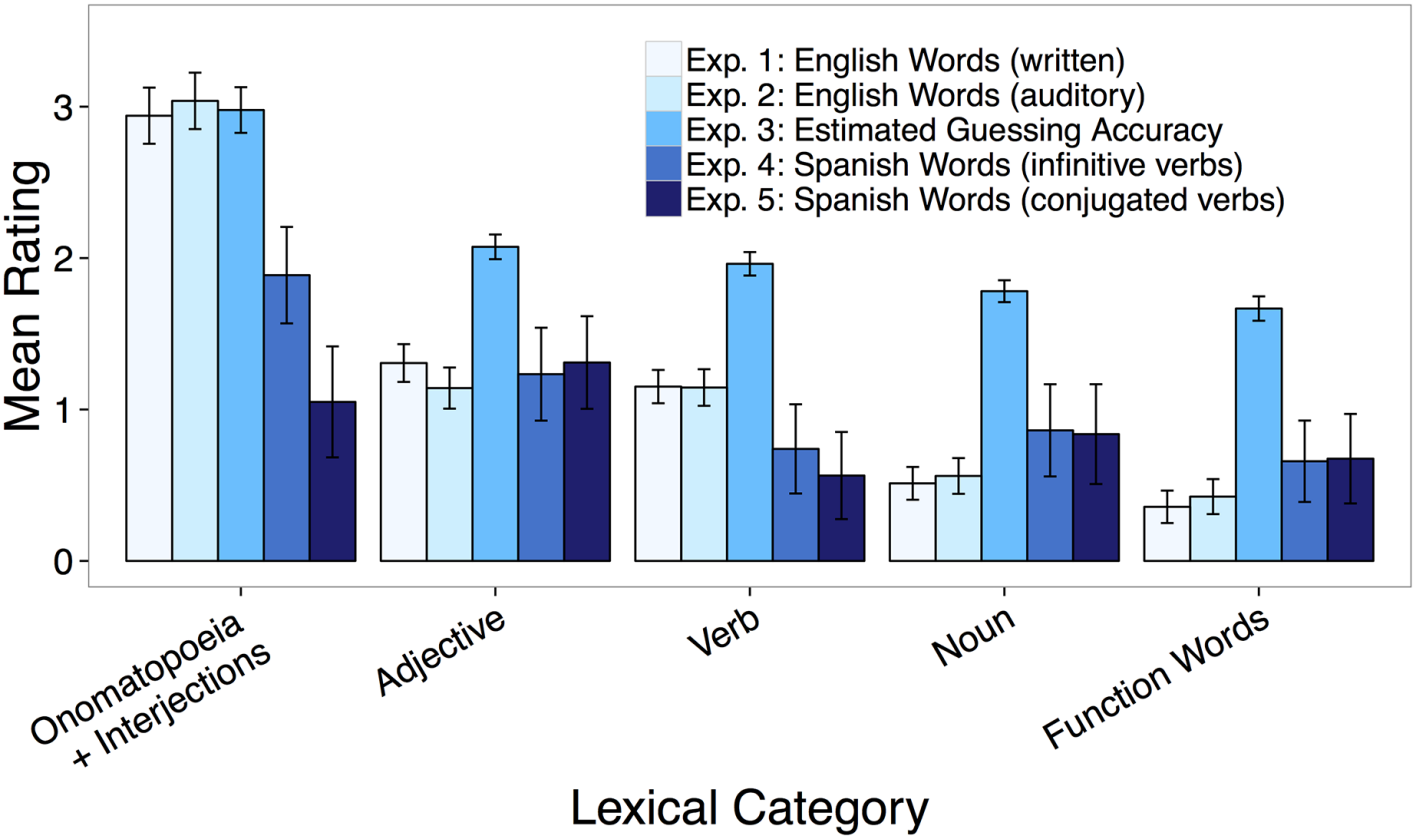
Relationship between words’ lexical category and iconicity ratings based on written and auditory English stimuli. estimates of an alien’s accuracy in guessing an English word’s meaning from its sound, iconicity ratings of written Spanish stimuli with verbs in infinitive form, and in 3^rd^ person singular form. The measure of guessing accuracy was rescaled to match the scale used in the other experiments for graphing purposes only. Error bars represent standard error of means.

The average rating across all words was .75, (SD = .99, range = -2.10–4.36), significantly different from 0, *t*(591) = 18.38, *p≪*.0001, indicating that, on average, the words were viewed to be mildly resembling their meanings, that is, iconic. Examples of words and accompanying ratings are shown in Table 1. The results for each word in Experiments 1–3 are presented in S1 Table. The iconicity ratings did not reflect a general bias to rate words as iconic, but as shown in Fig 1, the ratings varied by lexical category. Participants rated onomatopoeia, *M =* 3.15, and interjections, *M =* 2.70, as more iconic than all other lexical categories, *p≪*.0001. Adjectives, *M =* 1.31, and verbs, *M =* 1.15, were rated as more iconic than nouns, *M =* .51, and function words, *M =* .36, *p≪*.0001 (see Methods and S1 Results sections for description of all regression models).

**Table 1:** Examples of words with ratings from Experiment 1 from iconic (5) to opposite (-5) meanings.

Word	Lexical category	Average Rating
Moo	Onomatopoeia	3.88
Ouch	Interjection	3.46
Sticky	Adjective	2.93
Stop	Verb	2.50
Jeans	Noun	0.00
Here	Function Word	-0.20

To investigate the relationship between iconicity and AoA, we predicted iconicity from AoA. To rule out the contribution of plausible confounds, e.g., the possibility that short or frequent words are learned sooner and also tend to be more iconic, we partialed out the number of phonemes, number of morphemes, log frequency, concreteness, and the extent to which each word was associated with babies (see Methods for details). Controlling for all these properties, AoA was a strong predictor of iconicity, *b* = .006, 95% CI [.003, .008], *X* ^2^(1) = 20.19, *p≪*.0001 (see also S1A Fig). This effect continued to be reliable when systematicity [30] was added as a predictor, *b* = .004, 95% CI [.0005, .008], *X* ^2^(1) = 4.87, *p* = .03 (even though this meant reducing the sample size to the 294 words for which systematicity measures were available). AoA remained a reliable predictor of iconicity after removing the contribution of onomatopoeia and interjections, *b* = .003, 95% CI [.001, .006], *X* ^2^(1) = 7.50, *p* = .006, and after removing all 70 multimorphemic words, *b* = .006, 95% CI [.003, .008], *X* ^2^(1) = 19.05, *p≪*.0001.

## Experiment 2: English Words (auditory presentation)

In Experiment 2, we sought to replicate the main results of Experiment 1 using auditorily presented words. Rather than being asked to say the words aloud before making their ratings, participants listened to audio recordings of the same words spoken in a neutral tone of voice by a native speaker of American-English. The word was also presented in its written form to disambiguate homophones,

### Results

The average iconicity rating across all words was.78 (SD = .98, range = -2.18–4.64). This mean was significantly different from 0, *t*(589) = 19.21, *p≪*.0001, indicating mild overall iconicity. This mean was not reliably different from that in Experiment 1, *X* ^2^(1) = .31, *p* = .58. There was a moderate correlation between the ratings obtained in Experiment 1 and 2, *r =* .61, *p <* .0001. Shown in Fig 1, iconicity ratings varied by lexical category. Onomatopoeia, *M =* 3.39, and interjections, *M =* 2.46 (combined), were again rated as most iconic, *p*≪.0001. Adjectives, *M =* 1.14, and verbs, *M =* 1.15 were rated as more iconic than nouns, *M =* .56, and function words, *M =* .42, *p*≪.0001.

**Fig 2:**
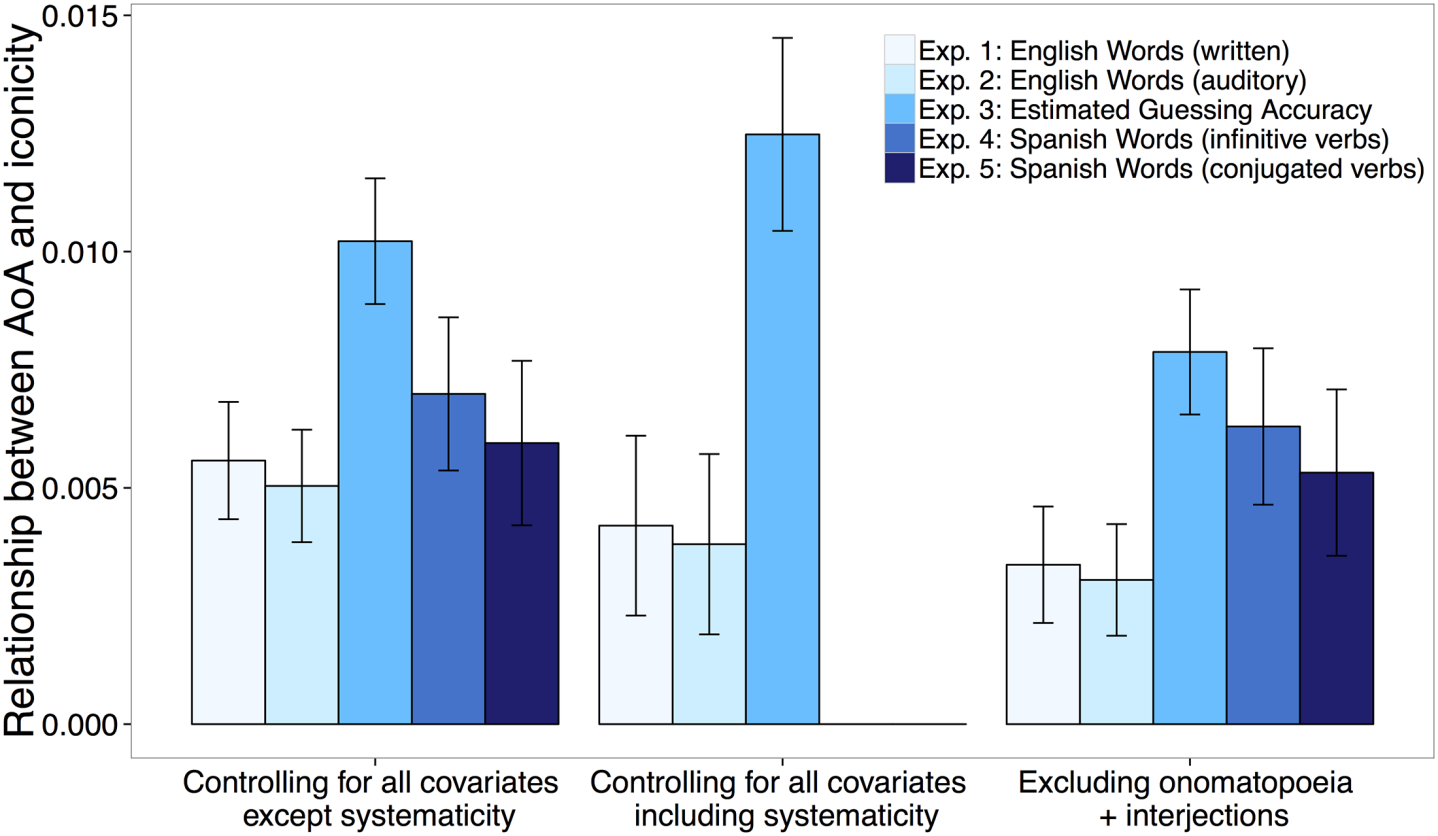
Relationship between AoA and iconicity ratings as measured by coefficients from each mixed effects regression model from each experiment. The coefficient represents increases in iconicity per each 1 percent increase in proportion of children that produce a given word at 30 months. The left-most bars depict coefficients from analyses controlling for effects of the basic set of covariates: frequency, number of phonemes, number of morphemes, concreteness, and association with babies for English language Experiments 1–3; frequency, number of phonemes and number of morphemes for Spanish language Experiments 4–5. The middle group of bars depict coefficients from analyses controlling for the basic set of covariates plus systematicity. The right-most bars depict coefficients from analyses controlling for the basic set of covariates and excluding all onomatopoeia and interjections. Estimates of alien accuracy were rescaled to match the scale used in the other experiments for graphing purposes only. Error bars represent standard error of means. *** indicates *p <* .0001, ** indicates *p <* .01, * indicates *p <* .05.

As shown in Fig 2, words learned earlier tended to be more iconic, *b* = .005, 95% CI [.003, .007], *X* ^2^(1) = 17.93, *p*≪.0001 (see also S1B Fig). This effect held for the reduced set of words for which we had systematicity measures, *b* = .004, 95% CI [.00007, .008], *X* ^2^(1) = 3.98, *p* = .05. AoA was still a reliable predictor of iconicity after removing onomatopoeia and interjections, *b* = .003, 95% CI [.0007, .005], *X* ^2^(1) = 6.68, *p* = .01.

## Experiment 3: English words (estimated guessing accuracy)

In Experiment 3, we sought to replicate the findings of the first two experiments using a more implicit task as an alternate measure of iconicity. Rather than asking people to directly rate the iconicity of each word, we asked participants to estimate how accurately (on a 0–100 scale) a “space alien” could guess the meaning of each word based only on its sound. As in Experiment 1, participants read the words and were instructed to speak them aloud before making their judgments.

### Results

The average judgment of accuracy across all words was 37.06 (SD = 11.97, range = 8.64–75.08). There was a moderate correlation between the estimates of guessing accuracy and the iconicity ratings from Experiment 1, *r =* .46, *p <* .0001. As shown in Fig 1, estimates of guessing accuracy varied with lexical category. Onomatopoeia, *M =* 56.55, and interjections, *M =* 63.15 (combined), were rated as most likely to be guessed accurately, *p≪*.0001. Adjectives, *M =* 41.48, and verbs, *M =* 39.24, were rated as more likely to be guessed accurately than nouns, *M =* 35.62, and function words, *M =* 33.33, all *p* < .05.

As shown in Fig 2, participants’ judgments of how likely an alien would guess a given word correlated with children’s AoA of those words, *b* = .10, 95% CI [.08, .13], *X* ^2^(1) = 58.81, *p*≪.0001 (see S1C Fig). The effect also held for the reduced set of words for which we had systematicity measures, *b* = .12, 95% CI [.08, .16], *X* ^2^(1) = 37.17, *p*≪.001. AoA was still a reliable predictor of participants’ estimation of guessing accuracy after removing onomatopoeia and interjections, *b* = .08, 95% CI [.05, .10], *X* ^2^(1) = 35.35, *p≪*.0001.

### Summary of Experiments 1–3

Across three experiments, participants rated the iconicity of 592 English words from the MCDI. In each experiment, we found the same pattern of results: iconicity varied with the lexical category and age of acquisition of words. Onomatopoeia and interjections were rated as more iconic than adjectives and verbs, which were rated as more iconic than nouns and function words. Words learned earliest by children were words that participants thought sounded like what they meant (Experiments 1–2) and words that would be most successfully guessed by an “alien” learner of English based on the way the word sounded (Experiment 3).

## Experiment 4: Spanish Words (infinitive verbs)

In Experiments 1–3 we showed that iconicity extends across English vocabulary varying systematically with lexical category and age of acquisition. It remains unclear whether these results are specific to English or generalize more widely. In Experiment 4 we asked native Spanish speakers to judge iconicity in 637 Spanish words from the MCDI, including 19 onomatopoetic words (11) and interjections (8), 60 adjectives, 102 verbs, 356 nouns, and 100 function words. We assessed whether Spanish exhibits the same basic patterns of iconicity to English with respect to lexical category and AoA. However, because Spanish is a verb-framed language and Spanish verbs are known to be less expressive of manner of movement, we predicted relatively lower iconicity ratings for verbs in Spanish as compared to English. As in Experiment 1, participants read the words and were instructed to speak them aloud before making their ratings.

### Results

The average judgment of iconicity across all words was .88 (SD = .97, range = -2.00–3.62). A t-test showed that the ratings were significantly different from 0, *t*(636) = 22.87, *p≪*.0001. The results for each word in Experiments 4–5 are presented in S2 Table. As in Experiments 1–3, judgments of iconicity varied with lexical category (Fig 1). Onomatopoeia, *M =* 1.72, and interjections, 2.06 (combined), and adjectives, *M =* 1.23, were rated higher in iconicity compared to nouns, *M =* .86, and function words, *M =* .64, and verbs, *M =* .74, all *p <* .05.

As shown in Fig 2, participants’ judgments of iconicity correlated with AoA, *b* = .007, 95% CI [.004, .01], *X* ^2^(1) = 18.56, *p*≪.0001 (the regression line is plotted in S1D Fig). AoA was still a reliable predictor of iconicity after removing onomatopoeia and interjections, *b* = .006, 95% CI [.003, .01], *X* ^2^(1) = 14.49, *p =* .0001.

## Experiment 5: Spanish Words (conjugated verbs)

As predicted, Spanish verbs were rated relatively low in iconicity compared to English verbs. However, the verbs were presented in their infinitive form, ending with a verb marker (–ar/-er/-ir). Potentially, the presence of this common suffix may have led participants to treat all the verbs in a similar way resulting in lower iconicity ratings. In Experiment 5 we replicated Experiment 4, but this time conjugated verbs into the third person singular form, e.g., tomar → toma.

### Results

The average judgment of accuracy across all words was .83 (SD = 1.03, range = -2.30–3.64), which was significantly different from 0, *t*(632) = 20.23, *p≪*.0001. This mean was not reliably different from that in Experiment 4, *X* ^2^(1) = .54, *p* = .46. The conjugated verbs were not rated differently from the infinitive verb forms presented in Experiment 4, *X* ^2^(1) = .42, *p =* .51. There was a moderate correlation between the iconicity ratings from Experiment 4 and Experiment 5, *r =* .41, *p* < .0001. As shown in Fig 1, judgments of iconicity varied with lexical category. Adjectives, *M =* 1.31, were rated as more iconic compared to nouns, *M =* .84, function words, *M =* .68, and verbs, *M =* .56, all *p <* .001. Interjections, *M =* 1.54, and onomatopoeia, M = .68 (combined) were rated no different than adjectives or nouns, but were rated marginally more iconic than function words, *p =* .08 and significantly more iconic than verbs, *p =* .03. The finding that onomatopoeia words were rated low in iconicity was unexpected given the results of the previous experiments. We discuss this finding further in the summary section below.

As shown in Fig 2, judgments of iconicity were again correlated with the Spanish AoA of those words, *b =* .006, 95% CI [.002, .008], *X* ^*2*^(1) = 11.67, *p =* .006 (see also S1E Fig). AoA was still a reliable predictor of iconicity after removing onomatopoeia and interjections, *b* = .005, 95% CI [.002, .009], *X* ^2^(1) = 9.14, *p =* .002.

To further test our claim that the observed relationship between AoA in iconicity is not artifactual, we took advantage of our having two measures of AoA and of iconicity—one for English, and one for Spanish. We identified 412 one-to-one English-Spanish translation pairs in the MCDI, and asked whether iconicity was better predicted by the AoA of the word in that particular language (e.g., English iconicity predicted specifically by *English* AoA).

**Fig 3:**
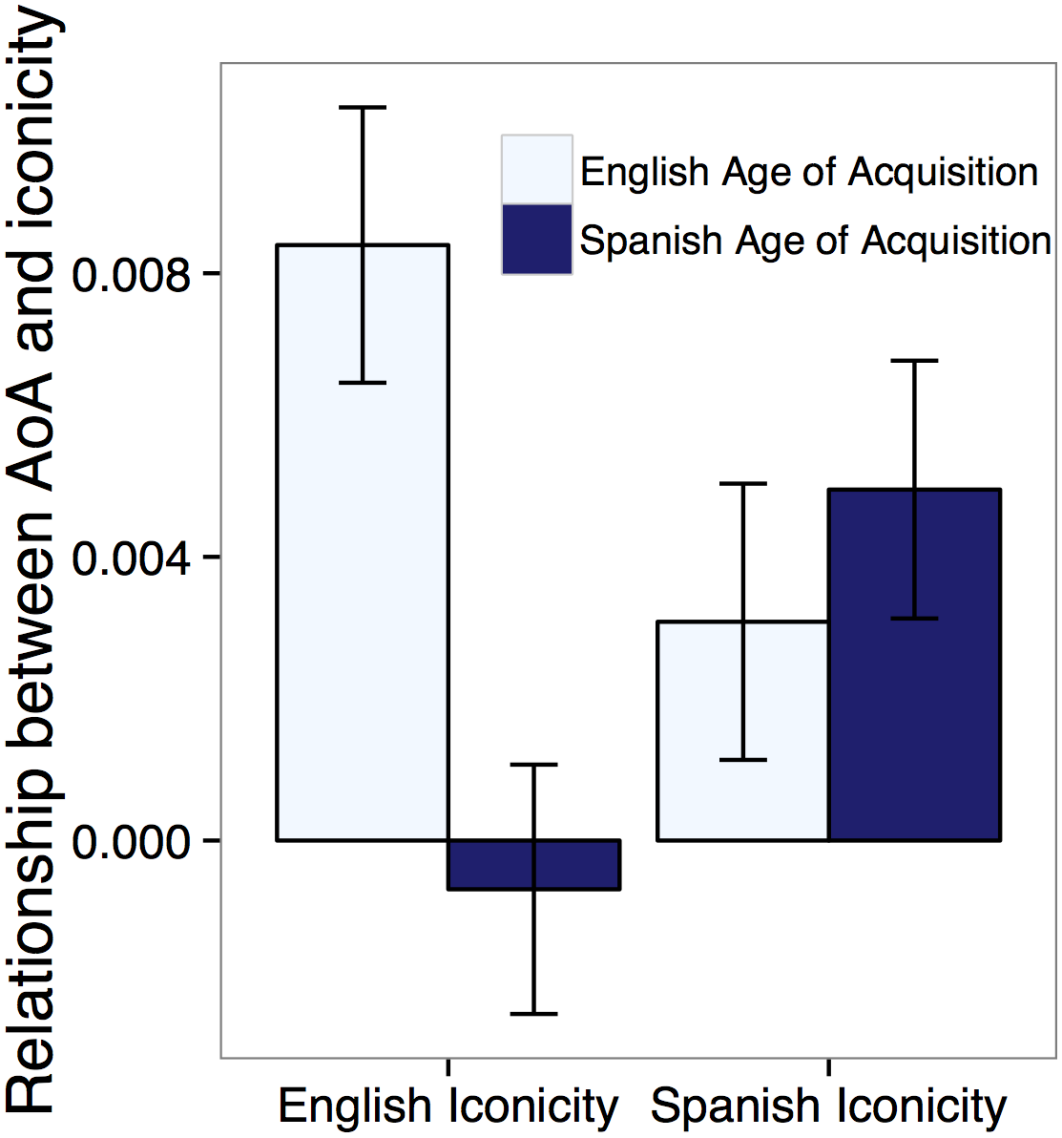
Relationship between AoA in each language and iconicity ratings in each language for translation pairs. The bars show the coefficient from a multiple regression predicting iconicity from AoA in each language. English iconicity ratings are based on the average judgments obtained from Experiment 1 (written words). Spanish iconicity ratings are based on the average judgments across Experiments 4 (infinitive verbs) and 5 (conjugated verbs). Error bars represent standard error of means.

Although there was a fairly strong correlation between English and Spanish AoA, *r* = .57, English AoA was a reliable predictor of English iconicity ratings, *b* = .008, 95% CI [.004, .012], *X* ^2^(1) = 18.7, *p*≪.001, but Spanish AoA was not, *X* ^2^(1) = .15, *p* = 0.70. Likewise, Spanish AoA remained a reliable predictor of Spanish iconicity ratings, *b* = .005, 95% CI [.001, .009], *X* ^2^(1) = 7.41, *p* < .01, but English AoA was not, *X* ^2^(1) = 2.51, *p* = 0.11 (see Fig 3). These results meant that differences in AoA between English and Spanish predicted the differences in iconicity, *b* = .007, 95% CI [.0006, .13], *t* = 2.15, *p* = .032. Words that were learned earlier in English compared to Spanish were more iconic in English than Spanish, and vice versa. This result serves as further evidence that the AoA-iconicity relationship is a real relationship rather than an artifact of an unexamined confound and highlight the language-specific nature of the relationship between AoA and iconicity for both English and Spanish.

### Summary of Experiments 4–5

In Experiments 4 and 5, native Spanish-speaking participants rated the iconicity of 632 Spanish words from the Spanish MCDI. As in English, ratings of iconicity of Spanish words correlated with Spanish AoA: words learned earlier tended to be rated as more iconic. When we compared the results from Spanish and English for the 412 one-to-one translation pairs, we found that the difference in AoA between the English and Spanish translation equivalents predicted the difference in iconicity: words that were learned earlier in English than Spanish tended to have higher iconicity values in English than Spanish (and vice versa). This analysis, in addition to the weak correlation found between the iconicity ratings of English-Spanish translations, helps to confirm that participants are attending to the *correspondence* between meaning and forms in their evaluations of iconicity rather than basing their judgments solely on the word meanings.

Also as in English, we found that participants in both Spanish experiments gave adjectives higher iconicity ratings than nouns and function words. Unlike in English, but in line with Spanish typology as a verb-framed language, participants rated verbs as relatively low in iconicity, comparable to nouns and function words. This pattern was found for verbs presented in both the infinitive and third person singular form.

An unexpected difference between English and Spanish was that onomatopoetic words in Spanish were rated quite low in iconicity. A likely reason is that onomatopoeia words listed on the MCDI may have been unfamiliar to many participants. Our participants were native Spanish speakers living within the United States, whereas the MCDI words were determined in part from Spanish learners in Mexico. Additionally, our Spanish translator suggested that some of the onomatopoeic words such as “ee”, the sound of a sheep, might not have been readily recognized outside the context of talking about animal sounds with children. To test this possibility, we asked 10 native Spanish-speaking participants on Amazon Mechanical Turk to define the onomatopoeic words. Indeed, for 5 of the 11 words, fewer than 25% of participants were able to provide accurate definitions.

## General Discussion

Indo-European languages in general, and English in particular, are often characterized as having highly arbitrary vocabularies [2,29]. We examined iconicity across roughly 600 of the earliest learned words in two Indo-European languages: English and Spanish. In both languages, native speakers rated words as being mildly iconic, on average.

In both English and Spanish, adjectives were rated as more iconic than nouns and function words, and in English, onomatopoeic words and interjections were rated most iconic of all (but not in Spanish, likely for reasons discussed above). Additionally, English verbs, which tend to express manner, were rated as relatively iconic, whereas Spanish verbs, which tend to lack manner information, were rated as less iconic. These results confirmed our predictions based on the large body of cross-linguistic research on ideophones and other iconic words in spoken languages [3,26]. They provide the most extensive evidence to date that iconicity is present in the vocabulary of spoken languages traditionally thought to be lacking in ideophonic forms.

The degree of iconicity across English and Spanish words was related to their age of acquisition. In all five experiments, earlier learned words were rated as more iconic. This relationship remained strong after accounting for several other factors known to relate to word learning, including frequency, concreteness, number of phonemes, and number of morphemes, and the extent to which each word was “associated with babies.” This latter control eliminates the possibility that our findings resulted from participants strategically rating baby-like words as more iconic. (Rating the words according to such a strategy would also not explain the way in which iconicity varied by the different lexical categories).

Although our work is, to our knowledge, the first to investigate iconicity across a large sample of vocabulary in spoken languages, past work [30,31] has examined *systematicity* in English, the extent to which differences in form correlate with difference in meaning. This work shows that there is a small but robust positive correlation such that similar sounding words tend to have similar meanings—an additional source of non-arbitrariness in language. Importantly, our findings hold when this measure of systematicity is partialed out of our iconicity ratings.

Our findings complement findings from British Sign Language showing that more iconic signs tend to be learned earlier [34,35], and they support the proposal that iconicity in the early vocabulary of languages serves to aid word learning [3,4]. Scholars have suggested that in spoken languages such as Japanese, which have a rich class of ideophones, caregivers may use these iconic words more frequently with infants and toddlers to provide them with an extra cue to link words to their meanings and generalize those meanings across different contexts [3]. Our results show that even in ideophone-impoverished languages like English and Spanish, iconicity concentrates in earlier learned words, and thus may still support the word learning process. An interesting question for future research is whether ideophone-rich languages exhibit the same relationship between iconicity and AoA across other word classes.

Altogether our results provide compelling new evidence that iconicity is alive and active within the English and Spanish vocabularies. Although often treated as binary, with a word or sign designated as either iconic or not, semiotic theories of iconicity have long observed that signals can exhibit varying degrees of iconicity [4, 43]. Indeed, we found evidence that iconicity in spoken languages is not categorical, wholly present in some words and absent in others, but rather, like in signed languages, it is graded and spread across the vocabulary. It is more concentrated in words that express sound and other sensorimotor properties, and also in the earliest learned words, cutting across lexical categories. However, a critical question remains: What are the mechanisms that would give rise to these patterns across languages? How might it come to be that in English, Spanish, and BSL, the words and signs that are learned earlier tend to be more iconic? And how might adjectives in English and Spanish come to be more iconic than nouns?

One possibility is that the relationship between iconicity and age of acquisition arises because iconicity facilitates word learning by children, and thus children tend to learn more iconic words earlier. However, such an explanation does not account for the differences in iconicity across lexical categories, which implicate historical processes in which the forms of words change differentially over time. This pattern of iconicity might arise if some words maintained a degree of iconicity after their original generation as iconic neologisms, or if they became more iconic over time. A plausible mechanism for development towards increased iconicity is the inclination of speakers to sometimes exaggerate the articulation of words in iconic ways, which over time could influence the standardized ways in which they are pronounced. For example, English speakers are known to extend the duration of a vowel to express temporal or spatial extension and to modulate the pitch of a word to express size or verticality, both modifications that affect vowel quality [26,44,45]. Moreover, research suggests that English speakers are especially likely to exaggerate iconic features of words during infant directed speech [46], which could contribute to a tendency for early learned vocabulary to become more iconic.

## Conclusion

Plato’s philosophical investigation into whether words are iconic or arbitrary cuts to an essential issue that has occupied scholars for millennia as we have sought to understand the nature of human language. The long history of investigation reveals that the answer to this question is complex. It is not sufficient to assert the blunt principle that words are arbitrary, nor is it constructive to simply point out iconic exceptions to arbitrariness. Instead, we must seek to understand the detailed ways that iconicity functions across languages [3,4,37].

The documentation of iconicity across signed and spoken languages is giving rise to a new domain of linguistic typology and historical investigation. Sign language scholars have been able to trace signs and grammatical constructions back to their iconic origins [11,47,48], and recent studies show that mature signed languages exhibit different typological tendencies in the use of iconicity [49]. In spoken languages, linguists are increasingly building a comparative and historical account of ideophone systems, including the processes by which new ideophones are generated [26,27,50]. Here we found that iconicity in spoken languages is not limited just to ideophone systems; rather it is distributed systematically across the vocabulary of even stereotypically arbitrary languages like English and Spanish.

## Methods

### Experiment 1

#### Participants

442 native English speakers were recruited from Amazon Mechanical Turk and received $0.35 for their participation. Participant recruitment was limited to the United States for all experiments. Participants in Experiments 1–3 all listed English as their native language. In all studies, judgments were collected until we had at least 10 ratings per word. The University of Wisconsin Madison Education and Social/Behavioral Science IRB approved a waiver of signed consent for these studies. Amazon Mechanical Turk does not allow participants ("workers") to provide identifying information to the experimenter ("requester"), therefore signed consent was not possible.

#### Stimuli

We used 592 words from the 680 words of the MacArthur-Bates Developmental Inventory of Words and Sentences, a normed list of the early productive vocabulary of 16-30-month-old toddlers learning American English [33]. This set of words included both monomorphemic (522) and multimorphemic words (70), but excluded all compound words and polysemous words with multiple entries. The list contained nouns (319), adjectives (60), verbs (99), onomatopoeia (10) and interjections (9), and function words (95). Function words comprised all closed class words including determiners, pronouns, question words, conjunctions, auxiliary verbs, prepositions and verb particles. The MCDI does not contain open class adverbs, such as those conveying manner of motion (e.g., in English, words productively derived from adjectives with the suffix–*ly*), as those are learned later in development. All words are listed with their lexical category in Table 1.

For statistical analyses, we combined onomatopoeia and interjections into a single grammatical category. Semantically, interjections are often characterized as conventionalized emotional exclamations (e.g., “uh oh”, “ouch”, “yum”) and in some cases, they are derived from highly scripted interactional routines (e.g., “hi”, “bye”). Syntactically and phonologically, interjections behave similarly to ideophones and onomatopoeia. They can constitute complete utterances on their own (i.e., they exhibit syntactic autonomy), and their phonology is often anomalous. Also like ideophones and onomatopoeia, scholars have noted an iconic quality of many interjections.

Our measure of age of acquisition (AoA) was the proportion of toddlers producing a given word at 30 months of age as based on norms from the CLEX database [51].

#### Procedure

We quantified iconicity of English words by collecting ratings from native English speakers, comparable to studies of signed languages [34,35]. We defined iconicity for our participants using the following set of instructions (note that the example of anti-iconicity comes from Hockett [2]: “‘Whale’ is a small word for a large object; ‘microorganism’ is the reverse,” and ‘dog’ and ‘cat’ are commonly used as examples of arbitrariness, as in Pinker and Bloom [52]):

*“Some English words sound like what they mean*. *For example*, *SLURP sounds like the noise made when you perform this kind of drinking action*. *An example that does not relate to the sound of an action is TEENY*, *which sounds like something very small (compared to HUGE which sounds big)*. *These words are* *iconic*. *You might be able to guess these words’ meanings even if you did not know English*. *Words can also sound like the* *opposite* *of what they mean*. *For example*, *MICROORGANISM is a large word that means something very small*. *And WHALE is a small word that means something very large*. *And finally*, *many words are not iconic or opposite at all*. *For example there is nothing canine or feline sounding about the words DOG or CAT*. *These words are* *arbitrary*. *If you did not know English*, *you would not be able to guess the meanings of these words*.*”*

Participants rated each word, one at a time, on a -5 to 5 scale ranging from “words that sound like the opposite of what they mean” (-5) to “words that sound like what they mean” (5). The 0 point corresponded to words that are arbitrary—“do not sound like what they mean or the opposite”. Each word was shown to the left of the scale. Participants were asked to say each word aloud before making their judgment. Each participant rated 20 words.

#### Analysis

We examined the relationship between iconicity and lexical category using linear mixed effects regression models (see S1 Results for full specification of the linear mixed effects models used in each experiment). Significance levels were calculated using chi-square tests that compared fit of mixed-effect models with and without the factor of interest [53].

We also examined the relationship between iconicity and AoA using linear mixed effects regression models. AoA is known to be correlated with a number of factors such as frequency (frequent words are learned earlier) and concreteness (concrete words are learned earlier). Included in the analyses were (log) frequency (based on American Nation Corpus; [54]), concreteness (based on MRC database; [55]), number of phonemes, number of morphemes, and Monaghan and colleagues’ systematicity measure [30]–a measure of statistical regularity of sound-meaning pairings.

Additionally, we sought to eliminate the possibility that raters had an existing bias to rate words perceived to be words commonly said by babies as more iconic. Such a bias could create a correlation between iconicity and AoA for reasons having nothing to do with the form of the word. To check whether people tended to have a bias to simply rate “baby words” as more iconic, we had a new group of 291 participants rate how much they associated each word with babies on a scale between 1 (not associated with babies) to 10 (very associated with babies) and regressed these ratings out of iconicity ratings as well.

Systematicity measures were only available for 294 of our 592 words [30]. Thus, we first conducted analyses on the full set of words by regressing out concreteness, log frequency, number of phonemes, number of morphemes from iconicity ratings, and association with babies, and then using AoA to predict the residuals. We then conducted a second analysis on the subset of words to examine whether AoA predicts iconicity even after accounting for systematicity. All of these models included random effects of subject (see S1 Results). See S1 Data for all data collected in Experiments 1–3.

### Experiment 2

#### Participants

343 native English speakers were recruited from Amazon Mechanical Turk and received $0.35 for their participation.

#### Stimuli

The stimuli were audio recordings of each word from Experiment 1 spoken by a female native English speaker. The speaker was naïve to the hypotheses of the study. Two words (“down”, “dinner”) were inadvertently not recorded and were therefore omitted from this experiment.

#### Procedure

The experimental and analysis procedures were identical to that of Experiment 1 except that participants listened to an audio recording of each word before making their judgment. The written word was still presented to ensure that participants interpreted the word accurately.

### Experiment 3

#### Participants

415 native English speakers were recruited from Amazon Mechanical Turk and received $0.35 for their participation.

#### Stimuli

The same words from Experiment 1 were used, except that “down” was inadvertently left out.

#### Procedure

Participants were told that a space alien who did not know any English was trying to translate English words into its own language. Participants were asked to judge how accurately the alien could guess each word’s meaning based only on how the word sounded. They responded on a scale from 0 to 100 with 0 indicating that the alien would likely guess the wrong meaning and 100 indicating that the alien would likely guess the correct meaning. Participants were asked to pronounce each word aloud before making their judgment. The analysis procedure was identical to that used in Experiments 1 and 2.

### Experiment 4

#### Participants

93 native Spanish speakers were recruited from Amazon Mechanical Turk and received $2.00 for their participation. Participants in Experiments 4–5 all listed Spanish as their native language. Because there are fewer Spanish-speaking than English-speaking Mechanical Turk workers in the United States, each participant had to complete more ratings (100) than in the English experiments (20), and we wanted to attract participants quickly. We therefore paid participants in Experiments 4 and 5 substantially more than the English-speaking participants in Experiments 1–3.

#### Stimuli

We used 637 words from the Spanish version of the MCDI Words and Sentences [56]. See S2 Table for the complete list of words with their lexical category. This set of words excluded all compound words and polysemous words with multiple entries. As in the English studies, we classified the words as nouns (356), adjectives (60), verbs (102), onomatopoeia (11) and interjections (8), and function words (100). The list contained one open class, manner adverb–“despacio.” Since our predictions for this class of word are most similar to adjectives, we included “despacio” with this class for analyses.

Our measure of AoA is the proportion of children learning Mexican-Spanish producing a given word at 30 months as based on norms from the CLEX database [51].

#### Procedure

The procedure was identical to that of Experiment 1, except all instructions, examples, and stimuli were presented in Spanish (see S1 Methods for Spanish language instructions) and participants each rated 100 words. The analysis procedure was similar to that of the previous studies. Here we regressed out (log) frequency [57], number of phonemes, and number of morphemes. See S2 Data for all data collected in Experiments 4–5.

### Experiment 5

#### Participants

73 native Spanish speakers were recruited from Amazon Mechanical Turk and received $2.00 for their participation.

#### Stimuli

We used the same stimuli as in Experiment 4, except all verbs were conjugated into the third person singular form rather than the infinitive form as they appear on the Spanish MCDI. Two verbs when conjugated into third person singular had identical forms to nouns on the list (cocina, nada), and so these words were not used.

#### Procedure

The procedure was identical to that of Experiment 4.

## Supporting Information

S1 Data Data collected in English Experiments 1–3. (CSV)

S2 Data Data collected in Spanish Experiments 4–5. (CSV)

S1 Fig Relationship between age of acquisition (as measured by proportion of children saying the word at 30 months of age) and A) iconicity ratings of written English words, B) iconicity ratings of auditorily-presented English words, C) estimates of an alien’s accuracy in guessing the meaning of English words from their sound, D) iconicity ratings of written Spanish words with verbs in infinitive form, and E) iconicity ratings of written Spanish words with verbs conjugated in the 3^rd^ person singular form. Error bands represent standard error of linear model estimates.
(TIFF)

S1 Methods Instructions used in Spanish Experiment 4 and 5. (DOCX)

S1 Results All analyses conducted for Experiments 1–5. (DOCX)

S1 Table Average iconicity ratings for all English words used in Experiments 1–3. (DOCX)

S2 Table Average iconicity ratings for all Spanish words used in Experiments 4–5. (DOCX)

## References

[1] Plato. Cratylus Reprinted with corr. Indianapolis: Hackett; 1999.

[2] Hockett CF. The origin of speech. Sci Am. 1960;203: 88–96. 14402211

[3] Imai M, Kita S. The sound symbolism bootstrapping hypothesis for language acquisition and language evolution. Philos Trans R Soc Lond B Biol Sci. 2014;369: 20130298 10.1098/rstb.2013.0298 25092666 PMC4123677

[4] Perniss P, Vigliocco G. The bridge of iconicity: from a world of experience to the experience of language. Philos Trans R Soc B Biol Sci. 2014;369: 20130300 10.1098/rstb.2013.0300 PMC4123679 25092668

[5] Taub SF. Language from the Body: Iconicity and Metaphor in American Sign Language. Cambridge University Press; 2001.

[6] Strickland B, Geraci C, Chemla E, Schlenker P, Kelepir M, Pfau R. Event representations constrain the structure of language: Sign language as a window into universally accessible linguistic biases. Proc Natl Acad Sci. 2015;112: 5968–5973. 10.1073/pnas.1423080112 25918419 PMC4434776

[7] Dingemanse M, Blasi DE, Lupyan G, Christiansen MH, Monaghan P. Arbitrariness, iconicity and systematicity in language. Trends Cogn Sci. in press; 10.1016/j.tics.2015.07.013 26412098

[8] Cuskley C, Kirby S. Synaesthesia, cross-modality and langauge evolution In: Simner J, Hubbard EM, editors. Oxford Handbook of Synaesthesia. Oxford: Oxford University Press; pp. 869–907.

[9] de Saussure F. Course in General Linguistics. La Salle, IL: Open Court; 1983.

[10] Hockett CF. In Search of Jove’s Brow. Am Speech. 1978;53: 243–313.

[11] Armstrong DF, Wilcox SE. The Gestural Origin of Language. 1 edition Oxford ; New York: Oxford University Press; 2007.

[12] Tomasello M. Origins of Human Communication. Cambridge, Mass: The MIT Press; 2008.

[13] Bentley M, Varon EJ. An accessory study of “phonetic symbolism”.Am J Psychol. 1933;45: 76–86. 10.2307/1414187

[14] Lupyan G, Casasanto D. Meaningless words promote meaningful categorization. Lang Cogn. 2015;7: 167–193. 10.1017/langcog.2014.21

[15] Ramachandran VS, Hubbard EM. Synaesthesia–a window into perception, thought and language. J Conscious Stud. 2001;8: 3–34.

[16] Cuskley C. Mappings between linguistic sound and motion. Public J Semiot. 2013;5: 39–62.

[17] Ahlner F, Zlatev J. Cross-Modal Iconicity. Sign Syst Stud. 2010;38: 298–346.

[18] Brown RW, Black AH, Horowitz AE. Phonetic symbolism in natural languages. J Abnorm Psychol. 1955;50: 388–393. 14381156 10.1037/h0046820

[19] Gebels G. An investigation of phonetic symbolism in different cultures. J Verbal Learn Verbal Behav. 1969;8: 310–312. 10.1016/S0022-5371(69)80083-6

[20] La Polla R. An experimental investigation into phonetic symbolism as it relates to Mandarin Chinese. Sound Symbolism. 1994.

[21] Berlin B. Evidence for pervasive synesthetic sound symbolism in ethnozoological nomencalture. Sound symbolism. 1994 pp. 76–93.

[22] Ultan R. Size-sound symbolism. Universals of Human Language. 1978.

[23] Tanz C. Sound Symbolism in Words Relating to Proximity and Distance. Lang Speech. 1971;14: 266–276. 5097642 10.1177/002383097101400307

[24] Urban M. Conventional sound symbolism in terms for organs of speech: A cross-linguistic study. Folia Linguist. 2011;45 10.1515/flin.2011.007

[25] Diffloth G. The notes on expressive meaning Papers from the Eigth Regional Meeting of Chicago Linguistic Society. Chicago: Chicago Linguistic Society; 1972 pp. 440–447.

[26] Dingemanse M. Advances in the Cross-Linguistic Study of Ideophones: Advances in the Cross-Linguistic Study of Ideophones. Lang Linguist Compass. 2012;6: 654–672. 10.1002/lnc3.361

[27] Voeltz EFK, Kilian-Hatz C. Ideophones. John Benjamins Publishing; 2001.

[28] Masuda K. The physical basis for phonological iconicity Insistent Images. John Benjamins Publishing; 2007 pp. 57–71.

[29] Vigliocco G, Perniss P, Vinson D. Language as a multimodal phenomenon: implications for language learning, processing and evolution. Philos Trans R Soc B Biol Sci. 2014;369: 20130292–20130292. 10.1098/rstb.2013.0292 PMC4123671 25092660

[30] Monaghan P, Shillcock RC, Christiansen MH, Kirby S. How arbitrary is language? Philos Trans R Soc B Biol Sci. 2014;369: 20130299–20130299. 10.1098/rstb.2013.0299 PMC4123678 25092667

[31] Farmer TA, Christiansen MH, Monaghan P. Phonological typicality influences on-line sentence comprehension. Proc Natl Acad Sci. 2006;103: 12203–12208. 10.1073/pnas.0602173103 16882728 PMC1567719

[32] Bergen BK. The Psychological Reality of Phonaesthemes. Language. 2004;80: 290–311. 10.1353/lan.2004.0056

[33] Fenson L, Dale PS, Reznick JS, Bates E, Thal DJ, Pethick SJ, et al Variability in Early Communicative Development. Monogr Soc Res Child Dev. 1994;59: i–185. 7845413

[34] Thompson RL, Vinson DP, Woll B, Vigliocco G. The Road to Language Learning Is Iconic Evidence From British Sign Language. Psychol Sci. 2012;23: 1443–1448. 10.1177/0956797612459763 23150275

[35] Vinson DP, Cormier K, Denmark T, Schembri A, Vigliocco G. The British Sign Language (BSL) norms for age of acquisition, familiarity, and iconicity. Behav Res Methods. 2008;40: 1079–1087. 10.3758/BRM.40.4.1079 19001399

[36] Nuckolls JB. To Be or Not To Be Ideophonically Impoverished. Proceedings of the Eleventh Annual Symposium about Language and Society—Austin. 2003. Available: http://studentorgs.utexas.edu/salsa/proceedings/2003/nuckolls.pdf

[37] Perlman M, Cain AA. Iconicity in Vocalization, Comparisons with Gesture, and Implications for Theories on the Evolution of Language. Gesture. in press;

[38] Beavers J, Levin B, Wei Tham S. The typology of motion expressions revisited. J Linguist. 2010;46: 331–377. 10.1017/S0022226709990272

[39] Nielsen A, Rendall D. The source and magnitude of sound-symbolic biases in processing artificial word material and their implications for language learning and transmission. Lang Cogn. 2012;4: 115–125. 10.1515/langcog-2012-0007

[40] Yoshida H. A Cross-Linguistic Study of Sound Symbolism in Children’s Verb Learning. J Cogn Dev. 2012;13: 232–265. 23807870 10.1080/15248372.2011.573515 PMC3691963

[41] Imai M, Kita S, Nagumo M, Okada H. Sound symbolism facilitates early verb learning. Cognition. 2008;109: 54–65. 10.1016/j.cognition.2008.07.015 18835600

[42] Kantartzis K, Imai M, Kita S. Japanese Sound-Symbolism Facilitates Word Learning in English-Speaking Children. Cogn Sci. 2011;35: 575–586. 10.1111/j.1551-6709.2010.01169.x

[43] Morris C. Signs, Language And Behavior Literary Licensing, LLC; 2011.

[44] Perlman M, Clark N, Johansson Falck M. Iconic prosody in story reading. Cogn Sci. 2014; 10.1111/cogs.12190 25351919

[45] Shintel H, Nusbaum HC, Okrent A. Analog acoustic expression in speech communication. J Mem Lang. 2006;55: 167–177. 10.1016/j.jml.2006.03.002

[46] Nygaard LC, Herold DS, Namy LL. The Semantics of Prosody: Acoustic and Perceptual Evidence of Prosodic Correlates to Word Meaning. Cogn Sci. 2009;33: 127–146. 10.1111/j.1551-6709.2008.01007.x 21585466

[47] Frishberg N. Arbitrariness and Iconicity: Historical Change in American Sign Language. Language. 1975;51: 696 10.2307/412894

[48] Wilcox SE. Symbol and symptom: Routes from gesture to signed langauge. Annu Rev Cogn Linguist. 2009;7: 89–110.

[49] Padden CA, Meir I, Hwang S-O, Lepic R, Seegers S, Sampson T. Patterned iconicity in sign language lexicons. Gesture. 2013;13: 287–308. 10.1075/gest.13.3.03pad

[50] Dingemanse M. Making new ideophones in Siwu: Creative depiction in conversation. Pragmat Soc. 2014;5: 384–405. 10.1075/ps.5.3.04din

[51] Jørgensen RN, Dale PS, Bleses D, Fenson L. CLEX: A cross-linguistic lexical norms database*. J Child Lang. 2010;37: 419–428. 10.1017/S0305000909009544 19570318

[52] Pinker S, Bloom P. Natural language and natural selection. Behav Brain Sci. 1990;13: 707–727. 10.1017/S0140525X00081061

[53] Baayen RH, Davidson DJ, Bates DM. Mixed-effects modeling with crossed random effects for subjects and items. J Mem Lang. 2008;59: 390–412. 10.1016/j.jml.2007.12.005

[54] Reppen R, Ide N, Suderman K. American National Corpus (ANC) Second Release [DVD]. Philadelphia; 2005.

[55] Wilson MD. The MRC Psycholinguistic Database: Machine Readable Dictionary, Version 2. Behav Res Methods Instruments Comput. 1988;20: 6–11.

[56] Jackson-Maldonado D, Thal DJ, Marchman VA, Newton T, Fenson L, Conboy B. MacArthur Inventarios del Desarrollo de Habilidades Comunicativas User’s Guide and Technical Manual. Baltimore: Brookes; 2003.

[57] Davies M. A Frequency Dictionary of Spanish: Core Vocabulary for Learners. Routledge; 2006.

