# Supplementary figures and images for: Iconicity in English and Spanish and Its Relation to Lexical Category and Age of Acquisition
Source: PLoS One. 2015 Sep 4;10(9):e0137147. doi: 10.1371/journal.pone.0137147 (PMC4560417; doi:10.1371/journal.pone.0137147)

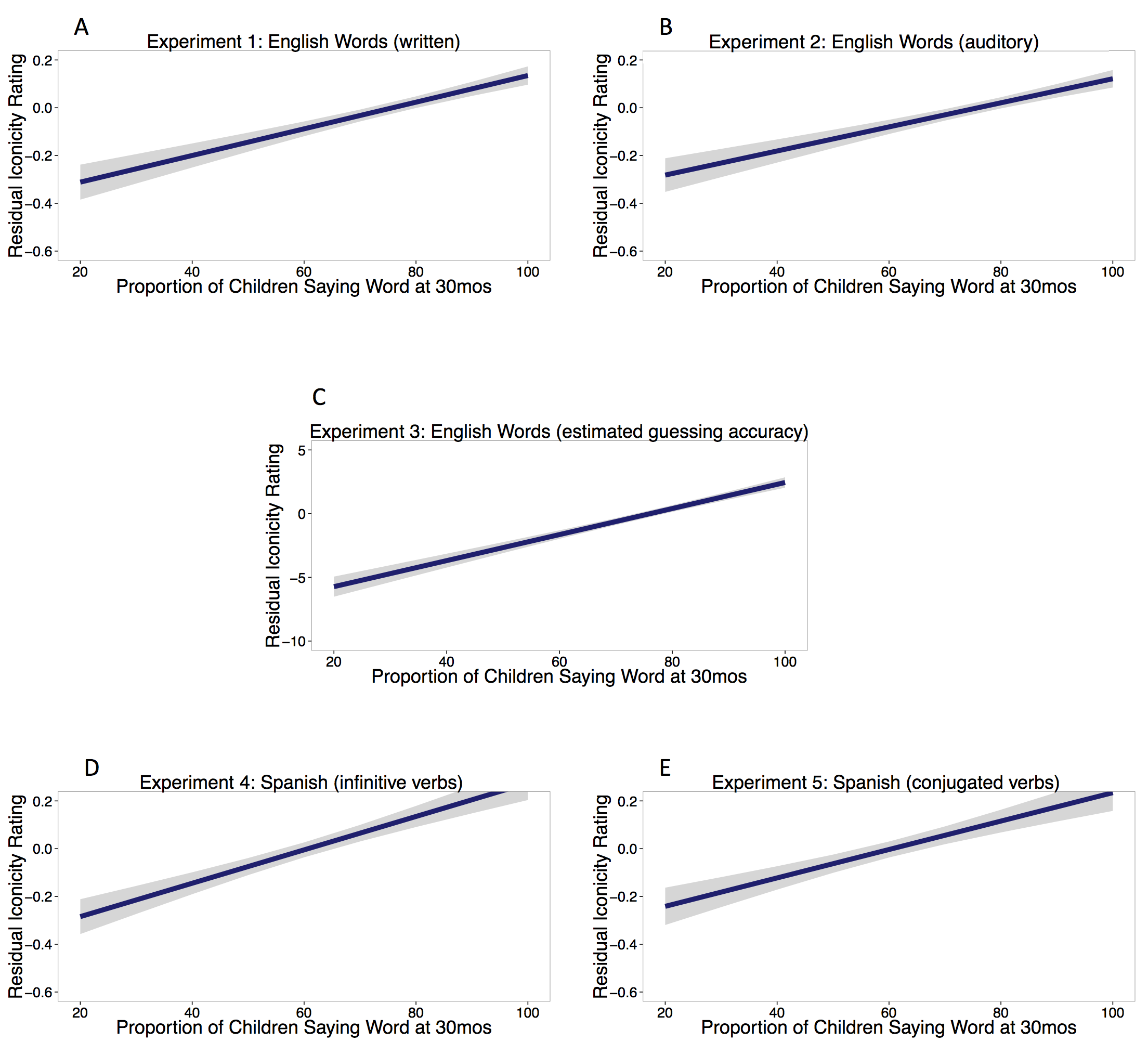

Supplement: S1 Fig — Error bands represent standard error of linear model estimates. (TIFF) [file pone.0137147.s003.tiff]
