# Supplementary material for: Iconicity in English and Spanish and Its Relation to Lexical Category and Age of Acquisition
Source: PLoS One. 2015 Sep 4;10(9):e0137147. doi: 10.1371/journal.pone.0137147 (PMC4560417; doi:10.1371/journal.pone.0137147)
Supplement: S1 Methods — (DOCX) [file pone.0137147.s004.docx]

**S1 Methods**

*Spanish instructions*

English instructions from Experiments 1 and 2 were translated into Spanish for Experiments 4 and 5 by a native speaker of Mexican Spanish. New language specific examples of highly iconic words and words with meanings opposite of their sounds were used when appropriate.

*Para este estudio, usted va a calificar 16 diferentes palabras en Español a base de cuanto cree usted que las palabras suenan como su significado. Déjenos explicarle acerca de esto. Algunas palabras en Español suenan como su significado. Por ejemplo, RONCAR suena como el ruido que se hace al respirar cuando alguien duerme. Un ejemplo que no tiene que ver con el sonido de una acción es CHICO, que suena como algo pequeño (comparado con GIGANTE que suena grande). Estas palabras son icónicas. Usted podría adivinar que significan estas palabras aunque no hablara el Español. Las palabras también a veces suenan como lo opuesto as su significado. Por ejemplo, MICROORGANISMO es una palabra grande que significa algo muy pequeño. Y MAR es una palabra pequeña que significa algo muy grande. Y finalmente, muchas palabras no son ni icónicas ni opuestas. Por ejemplo, no hay nada que suene canino o felino en las palabras PERRO y GATO. Estas palabras son arbitrarias. Si usted no supiera Español, no podría adivinar el significado de estas palabras.*

Prior to completing the ratings task, participants in Experiments 4-5 explained the meaning of two idiomatic Spanish phrases to verify that they could speak Spanish with native proficiency and were not using an online translator (which would provide a literal interpretation of the phrases). Participants who gave inappropriate answers for both phrases were excluded from data analyses.

¿Que significan las siguientes frases?

1) No me tomes el pelo.

*Idiomatic meaning: Don’t lie to me*

*Literal translation: don’t grab my hair*

2) No tiene pelos en la lengua

*Idiomatic meaning: He/she will say it like it is*

*Literal translation: He/she does not have hair on their tongue*
