# Supplementary material for: Iconicity in English and Spanish and Its Relation to Lexical Category and Age of Acquisition
Source: PLoS One. 2015 Sep 4;10(9):e0137147. doi: 10.1371/journal.pone.0137147 (PMC4560417; doi:10.1371/journal.pone.0137147)
Supplement: S1 Results — (DOCX) [file pone.0137147.s005.docx]

**S1 Results**

**Lexical category analyses**

rating = participants’ iconicity rating for each word

lexical category = to which lexical category a given word belongs (adjective, verb, noun, function words, or onomatopoeia and interjections)

All model comparisons were done on a subset of the data that included only the two lexical categories of interest. Then a model that included lexical category as a factor was compared to one that did not. We list the results of model comparisons for each individual analysis below, but all models had this structure:

a<-lmer (rating ~ lexicalCategory + (1|subjCode) + (1|word), data=d)

b<-lmer (rating ~ 1 + (1|subjCode) + (1|word), data=d)

**Experiment 1: English words (written)**

**Onomatopoeia and interjections versus adjectives**

Df AIC BIC logLik deviance Chisq Chi Df Pr(>Chisq)

b 4 4767.6 4787.4 -2379.8 4759.6

a 5 4736.1 4760.8 -2363.1 4726.1 33.491 1 7.16e-09 ***

A is the best fit model.

**Onomatopoeia and interjections versus verbs**

Df AIC BIC logLik deviance Chisq Chi Df Pr(>Chisq)

b 4 6963.8 6985.2 -3477.9 6955.8

a 5 6918.9 6945.7 -3454.5 6908.9 46.866 1 7.6e-12 ***

A is the best fit model.

**Onomatopoeia and interjections versus nouns**

Df AIC BIC logLik deviance Chisq Chi Df Pr(>Chisq)

b 4 19592 19617 -9791.8 19584

a 5 19439 19471 -9714.7 19429 154.26 1 < 2.2e-16 ***

A is the best fit model.

**Onomatopoeia and interjections versus function words**

Df AIC BIC logLik deviance Chisq Chi Df Pr(>Chisq)

b 4 6669.9 6691.1 -3331.0 6661.9

a 5 6573.4 6599.9 -3281.7 6563.4 98.57 1 < 2.2e-16 ***

A is the best fit model.

**Adjectives versus nouns**

Df AIC BIC logLik deviance Chisq Chi Df Pr(>Chisq)

b 4 21712 21738 -10852 21704

a 5 21668 21700 -10829 21658 46.28 1 1.025e-11 ***

A is the best fit model.

**Adjectives versus function words**

Df AIC BIC logLik deviance Chisq Chi Df Pr(>Chisq)

b 4 8874.3 8896.7 -4433.1 8866.3

a 5 8839.9 8867.9 -4414.9 8829.9 36.377 1 1.626e-09 ***

A is the best fit model.

**Verbs versus nouns**

Df AIC BIC logLik deviance Chisq Chi Df Pr(>Chisq)

b 4 23863 23889 -11927 23855

a 5 23825 23858 -11907 23815 40.003 1 2.535e-10 ***

A is the best fit model.

**Verbs versus function words**

Df AIC BIC logLik deviance Chisq Chi Df Pr(>Chisq)

b 4 11068 11091 -5529.9 11060

a 5 11038 11067 -5513.9 11028 31.913 1 1.612e-08 ***

A is the best fit model.

**Experiment 2: English words (spoken)**

**Onomatopoeia and interjections versus adjectives**

Df AIC BIC logLik deviance Chisq Chi Df Pr(>Chisq)

b 4 4556.9 4576.5 -2274.4 4548.9

a 5 4517.0 4541.5 -2253.5 4507.0 41.896 1 9.625e-11 ***

A is the best fit model.

**Onomatopoeia and interjections versus verbs**

Df AIC BIC logLik deviance Chisq Chi Df Pr(>Chisq)

b 4 6373.3 6394.4 -3182.6 6365.3

a 5 6333.5 6359.9 -3161.7 6323.5 41.778 1 1.022e-10 ***

A is the best fit model.

**Onomatopoeia and interjections versus nouns**

Df AIC BIC logLik deviance Chisq Chi Df Pr(>Chisq)

b 4 17707 17732 -8849.5 17699

a 5 17543 17575 -8766.5 17533 165.99 1 < 2.2e-16 ***

A is the best fit model.

**Onomatopoeia and interjections versus function words**

Df AIC BIC logLik deviance Chisq Chi Df Pr(>Chisq)

b 4 5986.2 6007.1 -2989.1 5978.2

a 5 5897.1 5923.2 -2943.5 5887.1 91.155 1 < 2.2e-16 ***

A is the best fit model.

**Adjectives versus nouns**

Df AIC BIC logLik deviance Chisq Chi Df Pr(>Chisq)

b 4 19777 19803 -9884.5 19769

a 5 19755 19788 -9872.6 19745 23.67 1 1.144e-06 ***

A is the best fit model.

**Adjectives versus function words**

Df AIC BIC logLik deviance Chisq Chi Df Pr(>Chisq)

b 4 8234.2 8256.4 -4113.1 8226.2

a 5 8213.0 8240.8 -4101.5 8203.0 23.126 1 1.517e-06 ***

A is the best fit model.

**Verbs versus nouns**

Df AIC BIC logLik deviance Chisq Chi Df Pr(>Chisq)

b 4 21535 21561 -10763 21527

a 5 21494 21526 -10742 21484 43.187 1 4.974e-11 ***

A is the best fit model.

**Verbs versus function words**

Df AIC BIC logLik deviance Chisq Chi Df Pr(>Chisq)

b 4 9978.8 10001.8 -4985.4 9970.8

a 5 9946.0 9974.9 -4968.0 9936.0 34.739 1 3.77e-09 ***

A is the best fit model.

**Experiment 3: English words (estimated guessing accuracy)**

**Onomatopoeia and interjections versus adjectives**

Df AIC BIC logLik deviance Chisq Chi Df Pr(>Chisq)

b 4 9964.9 9984.7 -4978.4 9956.9

a 5 9937.3 9962.1 -4963.7 9927.3 29.557 1 5.43e-08 ***

A is the best fit model.

**Onomatopoeia and interjections versus verbs**

Df AIC BIC logLik deviance Chisq Chi Df Pr(>Chisq)

b 4 14412 14434 -7202.2 14404

a 5 14380 14407 -7185.0 14370 34.291 1 4.746e-09 ***

A is the best fit model.

**Onomatopoeia and interjections versus nouns**

Df AIC BIC logLik deviance Chisq Chi Df Pr(>Chisq)

b 4 40662 40688 -20327 40654

a 5 40595 40627 -20292 40585 69.387 1 < 2.2e-16 ***

A is the best fit model.

**Onomatopoeia and interjections versus function words**

Df AIC BIC logLik deviance Chisq Chi Df Pr(>Chisq)

b 4 14034 14056 -7013.2 14026

a 5 13976 14003 -6983.1 13966 60.081 1 9.105e-15 ***

A is the best fit model.

**Adjectives versus nouns**

Df AIC BIC logLik deviance Chisq Chi Df Pr(>Chisq)

b 4 45348 45374 -22670 45340

a 5 45329 45361 -22659 45319 21.402 1 3.724e-06 ***

A is the best fit model.

**Adjectives versus function words**

Df AIC BIC logLik deviance Chisq Chi Df Pr(>Chisq)

b 4 18835 18858 -9413.6 18827

a 5 18825 18853 -9407.7 18815 11.886 1 0.0005656 ***

A is the best fit model.

**Verbs versus nouns**

Df AIC BIC logLik deviance Chisq Chi Df Pr(>Chisq)

b 4 49748 49774 -24870 49740

a 5 49733 49766 -24861 49723 17.248 1 3.28e-05 ***

A is the best fit model.

**Verbs versus function words**

Df AIC BIC logLik deviance Chisq Chi Df Pr(>Chisq)

b 4 23266 23290 -11629 23258

a 5 23264 23293 -11627 23254 4.7061 1 0.03006 *

A is the best fit model.

**Experiment 4: Spanish words (infinitive verbs)**

**Onomatopoeia and interjections versus adjectives**

Df AIC BIC logLik deviance Chisq Chi Df Pr(>Chisq)

b 4 5268.5 5288.5 -2630.3 5260.5

a 5 5269.4 5294.4 -2629.7 5259.4 1.146 1 0.2844

B is the best fit model. (No difference between lexical categories)

**Onomatopoeia and interjections versus verbs**

Df AIC BIC logLik deviance Chisq Chi Df Pr(>Chisq)

b 4 7511.3 7532.9 -3751.7 7503.3

a 5 7503.4 7530.3 -3746.7 7493.4 9.8887 1 0.001663 **

A is the best fit model.

**Onomatopoeia and interjections versus nouns**

Df AIC BIC logLik deviance Chisq Chi Df Pr(>Chisq)

b 4 22057 22083 -11025 22049

a 5 22055 22088 -11023 22045 4.0696 1 0.04366 *

A is the best fit model.

**Onomatopoeia and interjections versus function words**

Df AIC BIC logLik deviance Chisq Chi Df Pr(>Chisq)

b 4 7561.1 7582.7 -3776.6 7553.1

a 5 7552.6 7579.6 -3771.3 7542.6 10.518 1 0.001182 **

A is the best fit model.

**Adjectives versus nouns**

Df AIC BIC logLik deviance Chisq Chi Df Pr(>Chisq)

b 4 24045 24071 -12018 24037

a 5 24041 24074 -12016 24031 5.9082 1 0.01507 *

A is the best fit model.

**Adjectives versus function words**

Df AIC BIC logLik deviance Chisq Chi Df Pr(>Chisq)

b 4 9554.0 9576.5 -4773.0 9546.0

a 5 9536.5 9564.6 -4763.2 9526.5 19.584 1 9.63e-06 ***

A is the best fit model.

**Verbs versus nouns**

Df AIC BIC logLik deviance Chisq Chi Df Pr(>Chisq)

b 4 26236 26263 -13114 26228

a 5 26236 26269 -13113 26226 2.2895 1 0.1303

B is the best fit model. (No difference between lexical categories.)

**Verbs versus function words**

Df AIC BIC logLik deviance Chisq Chi Df Pr(>Chisq)

b 4 11707 11730 -5849.3 11699

a 5 11708 11738 -5849.1 11698 0.4276 1 0.5132

A is the best fit model.

**Experiment 5: Spanish words (conjugated verbs)**

**Onomatopoeia and interjections versus adjectives**

Df AIC BIC logLik deviance Chisq Chi Df Pr(>Chisq)

b 4 4331.1 4350.4 -2161.6 4323.1

a 5 4332.7 4356.8 -2161.4 4322.7 0.3902 1 0.5322

B is the best fit model. (No difference between lexical categories).

**Onomatopoeia and interjections versus verbs**

Df AIC BIC logLik deviance Chisq Chi Df Pr(>Chisq)

b 4 6374.6 6395.5 -3183.3 6366.6

a 5 6371.8 6397.9 -3180.9 6361.8 4.7627 1 0.02908 *

A is the best fit model.

**Onomatopoeia and interjections versus nouns**

Df AIC BIC logLik deviance Chisq Chi Df Pr(>Chisq)

b 4 20640 20666 -10316 20632

a 5 20641 20673 -10316 20631 0.8882 1 0.346

B is the best fit model. (No difference between lexical categories).

**Onomatopoeia and interjections versus function words**

Df AIC BIC logLik deviance Chisq Chi Df Pr(>Chisq)

b 4 6266.4 6287.3 -3129.2 6258.4

a 5 6265.3 6291.4 -3127.7 6255.3 3.0972 1 0.07843 .

A is the best fit model.

**Adjectives versus nouns**

Df AIC BIC logLik deviance Chisq Chi Df Pr(>Chisq)

b 4 22736 22762 -11364 22728

a 5 22726 22758 -11358 22716 11.983 1 0.0005367 ***

A is the best fit model.

**Adjectives versus function words**

Df AIC BIC logLik deviance Chisq Chi Df Pr(>Chisq)

b 4 8452.4 8474.5 -4222.2 8444.4

a 5 8435.6 8463.2 -4212.8 8425.6 18.804 1 1.449e-05 ***

A is the best fit model.

**Verbs versus nouns**

Df AIC BIC logLik deviance Chisq Chi Df Pr(>Chisq)

b 4 24802 24829 -12397 24794

a 5 24799 24832 -12394 24789 5.7365 1 0.01662 *

A is the best fit model.

**Verbs versus function words**

Df AIC BIC logLik deviance Chisq Chi Df Pr(>Chisq)

b 4 10374 10398 -5183.3 10366

a 5 10376 10405 -5183.1 10366 0.4491 1 0.5027

B is the best fit model. (No difference between lexical categories).

**Age of acquisition analyses**

logFreq = log frequency of each word

concreteness = concreteness rating of each word

phonemes = number of phonemes in each word

totalMorphemes = number of phonemes in each word

babyAVG = average babyness rating for each word

X30mos = proportion of children producing each word at 30 months

systematicity = systematicity rating for each word

**Experiment 1: English words (written)**

**1) Relationship between iconicity and age of acquisition:**

w$residRating = resid (lmer (rating ~ logFreq + concreteness + phonemes + totalMorphemes + babyAVG + (1|subjCode), data = w, na.action = na.exclude))

a <- lmer(residRating ~ X30mos + (1|subjCode),data=w)

b <- lmer(residRating ~ 1 + (1|subjCode),data=w)

Df AIC BIC logLik deviance Chisq Chi Df Pr(>Chisq)

b 3 31795 31815 -15894 31789

a 4 31776 31804 -15884 31768 20.19 1 7.011e-06 ***

A is the best fit model

**2) Relationship between iconicity and age of acquisition factoring out systematicity:**

w$residRating = resid (lmer (rating ~ logFreq + concreteness + phonemes + totalMorphemes + babyAVG + systematicity+ (1|subjCode), data = w, na.action = na.exclude))

a <- lmer(residRating ~ X30mos + (1|subjCode),data=w)

b <- lmer(residRating ~ 1 + (1|subjCode),data=w)

Df AIC BIC logLik deviance Chisq Chi Df Pr(>Chisq)

b 3 15865 15883 -7929.3 15859

a 4 15862 15887 -7926.9 15854 4.8714 1 0.02731 *

A is the best fit model

**3) Relationship between iconicity and age of acquisition excluding onomatopoeia and interjections:**

w<-subset(w, lexicalCategory!= " onomatopoeia and interjections")

w$residRating = resid (lmer (rating ~ logFreq + concreteness + phonemes + totalMorphemes + babyAVG + (1|subjCode), data = w, na.action = na.exclude))

a <- lmer(residRating ~ X30mos + (1|subjCode),data=w)

b <- lmer(residRating ~ 1 + (1|subjCode),data=w)

Df AIC BIC logLik deviance Chisq Chi Df Pr(>Chisq)

b 3 30879 30900 -15437 30873

a 4 30874 30901 -15433 30866 7.4997 1 0.006171 **

A is the best fit model

**4) Relationship between iconicity and age of acquisition excluding multimorphemic words:**

w<-subset(w, totalMorphemes==1)

w$residRating = resid (lmer (rating ~ logFreq + concreteness + phonemes + totalMorphemes + babyAVG + (1|subjCode), data = w, na.action = na.exclude))

a <- lmer(residRating ~ X30mos + (1|subjCode),data=w)

b <- lmer(residRating ~ 1 + (1|subjCode),data=w)

Df AIC BIC logLik deviance Chisq Chi Df Pr(>Chisq)

b 3 28018 28038 -14006 28012

a 4 28001 28028 -13996 27993 19.047 1 1.276e-05 ***

A is the best fit model

**Experiment 2: English words (spoken)**

**1) relationship between iconicity and age of acquisition:**

s$residRating = resid (lmer (rating ~ logFreq + concreteness + phonemes + totalMorphemes + babyAVG + (1|subjCode), data = s, na.action = na.exclude))

a <- lmer(residRating ~ X30mos + (1|subjCode),data=s)

b <- lmer(residRating ~ 1 + (1|subjCode),data=s)

Df AIC BIC logLik deviance Chisq Chi Df Pr(>Chisq)

b 3 28881 28901 -14437 28875

a 4 28865 28892 -14428 28857 17.932 1 2.289e-05 ***

A is the best fit model.

**2) Relationship between iconicity and age of acquisition factoring out systematicity:**

s$residRating = resid (lmer (rating ~ logFreq + concreteness + phonemes + totalMorphemes + babyAVG + systematicity+ (1|subjCode), data = s, na.action = na.exclude))

a <- lmer(residRating ~ X30mos + (1|subjCode),data=s)

b <- lmer(residRating ~ 1 + (1|subjCode),data=s)

Df AIC BIC logLik deviance Chisq Chi Df Pr(>Chisq)

b 3 14541 14560 -7267.7 14535

a 4 14540 14564 -7265.7 14532 3.9838 1 0.04594 *

A is the best fit model.

**3) Relationship between iconicity and age of acquisition excluding onomatopoeia and interjections:**

s<-subset(s, lexicalCategory!= " onomatopoeia and interjections")

s$residRating = resid (lmer (rating ~ logFreq + concreteness + phonemes + totalMorphemes + babyAVG + (1|subjCode), data = s, na.action = na.exclude))

a <- lmer(residRating ~ X30mos + (1|subjCode),data=s)

b <- lmer(residRating ~ 1 + (1|subjCode),data=s)

Df AIC BIC logLik deviance Chisq Chi Df Pr(>Chisq)

b 3 28089 28109 -14041 28083

a 4 28084 28111 -14038 28076 6.6764 1 0.00977 **

A is the best fit model.

**Experiment 3:** **English words (estimated guessing accuracy)**

**1) Relationship between iconicity and age of acquisition:**

al$residRating = resid (lmer (rating ~ logFreq + concreteness + phonemes + totalMorphemes + babyAVG + (1|subjCode), data = al, na.action = na.exclude))

a <- lmer(residRating ~ X30mos + (1|subjCode),data=al)

b <- lmer(residRating ~ 1 + (1|subjCode),data=al)

Df AIC BIC logLik deviance Chisq Chi Df Pr(>Chisq)

b 3 66855 66875 -33424 66849

a 4 66798 66826 -33395 66790 58.813 1 1.734e-14 ***

A is the best fit model

**2) Relationship between iconicity and age of acquisition factoring out systematicity:**

al$residRating = resid (lmer (rating ~ logFreq + concreteness + phonemes + totalMorphemes + babyAVG + systematicity+ (1|subjCode), data = al, na.action = na.exclude))

a <- lmer(residRating ~ X30mos + (1|subjCode),data=al)

b <- lmer(residRating ~ 1 + (1|subjCode),data=al)

Df AIC BIC logLik deviance Chisq Chi Df Pr(>Chisq)

b 3 33186 33204 -16590 33180

a 4 33150 33175 -16571 33142 37.173 1 1.081e-09 ***

A is the best fit model

**3) Relationship between iconicity and age of acquisition excluding onomatopoeia and interjections:**

al<-subset(al, lexicalCategory!= " onomatopoeia and interjections")

al$residRating = resid (lmer (rating ~ logFreq + concreteness + phonemes + totalMorphemes + babyAVG + (1|subjCode), data = al, na.action = na.exclude))

a <- lmer(residRating ~ X30mos + (1|subjCode),data=al)

b <- lmer(residRating ~ 1 + (1|subjCode),data=al)

Df AIC BIC logLik deviance Chisq Chi Df Pr(>Chisq)

b 3 65333 65353 -32663 65327

a 4 65299 65327 -32646 65291 35.345 1 2.761e-09 ***

A is the best fit model

**Experiment 4: Spanish words (infinitive verbs)**

**1) Relationship between iconicity and age of acquisition:**

i$residRating = resid (lmer (rating ~ logFreq + phonemes + totalMorphemes + (1|subjCode), data = i, na.action = na.exclude))

a <- lmer(residRating ~ X30mos + (1|subjCode),data=i)

b <- lmer(residRating ~ 1 + (1|subjCode),data=i)

Df AIC BIC logLik deviance Chisq Chi Df Pr(>Chisq)

b 3 26336 26356 -13165 26330

a 4 26319 26346 -13156 26311 18.56 1 1.647e-05 ***

A is the best fit model

**2) Relationship between iconicity and age of acquisition excluding onomatopoeia and interjections:**

i<-subset(i, lexicalCategory!= " onomatopoeia and interjections")

i$residRating = resid (lmer (rating ~ logFreq + phonemes + totalMorphemes + (1|subjCode), data = i, na.action = na.exclude))

a <- lmer(residRating ~ X30mos + (1|subjCode),data=i)

b <- lmer(residRating ~ 1 + (1|subjCode),data=i)

Df AIC BIC logLik deviance Chisq Chi Df Pr(>Chisq)

b 3 25839 25859 -12916 25833

a 4 25827 25853 -12909 25819 14.479 1 0.0001417 ***

A is the best fit model

**Experiment 5: Spanish words (conjugated verbs)**

**1) Relationship between iconicity and age of acquisition:**

c$residRating = resid (lmer (rating ~ logFreq + phonemes + totalMorphemes + (1|subjCode), data = c, na.action = na.exclude))

a <- lmer(residRating ~ X30mos + (1|subjCode),data=c)

b <- lmer(residRating ~ 1 + (1|subjCode),data=c)

Df AIC BIC logLik deviance Chisq Chi Df Pr(>Chisq)

b 3 23881 23901 -11938 23875

a 4 23872 23898 -11932 23864 11.667 1 0.0006361 ***

A is the best fit model

**2) Relationship between iconicity and age of acquisition excluding onomatopoeia and interjections:**

c<-subset(c, lexicalCategory!= " onomatopoeia and interjections")

c$residRating = resid (lmer (rating ~ logFreq + phonemes + totalMorphemes + (1|subjCode), data = c, na.action = na.exclude))

a <- lmer(residRating ~ X30mos + (1|subjCode),data=c)

b <- lmer(residRating ~ 1 + (1|subjCode),data=c)

Df AIC BIC logLik deviance Chisq Chi Df Pr(>Chisq)

b 3 23627 23646 -11810 23621

a 4 23620 23646 -11806 23612 9.1432 1 0.002496 **

A is the best fit model
