# Supplementary material for: Iconicity in English and Spanish and Its Relation to Lexical Category and Age of Acquisition
Source: PLoS One. 2015 Sep 4;10(9):e0137147. doi: 10.1371/journal.pone.0137147 (PMC4560417; doi:10.1371/journal.pone.0137147)
Supplement: S1 Table — (DOCX) [file pone.0137147.s006.docx]

S1 Table: English words used in Experiments 1-3.

| **Lexical Category** | **English word** | **Experiment 1 (written words)** | **Experiment 2 (spoken words)** | **Experiment 3 (estimated guessing accuracy)** |
| --- | --- | --- | --- | --- |
| adjective | asleep | 0.45 | 0.30 | 44.11 |
| adjective | awake | 2.10 | 1.83 | 37.50 |
| adjective | bad | 0.56 | 0.70 | 50.60 |
| adjective | better | -0.10 | 0.79 | 32.56 |
| adjective | big | 1.09 | -0.91 | 39.67 |
| adjective | black | 1.19 | 1.50 | 34.82 |
| adjective | blue | -0.07 | -0.40 | 45.42 |
| adjective | broken | 1.50 | 1.77 | 40.21 |
| adjective | brown | 0.57 | -0.30 | 22.17 |
| adjective | cold | 1.50 | 1.94 | 38.20 |
| adjective | cute | 2.08 | 2.13 | 38.55 |
| adjective | dark | 1.18 | 1.47 | 46.47 |
| adjective | dirty | 1.00 | 2.18 | 42.92 |
| adjective | empty | 0.80 | 1.40 | 42.60 |
| adjective | fast | 2.17 | 0.79 | 44.20 |
| adjective | fine | -0.46 | 0.30 | 31.50 |
| adjective | first | 1.30 | 0.50 | 28.67 |
| adjective | full | 1.79 | 0.71 | 43.93 |
| adjective | gentle | 2.64 | 0.71 | 36.33 |
| adjective | good | 0.93 | 0.82 | 48.30 |
| adjective | green | 1.10 | 0.82 | 37.60 |
| adjective | happy | 2.08 | 0.91 | 50.20 |
| adjective | hard | 3.69 | 3.00 | 45.00 |
| adjective | heavy | 1.10 | 0.91 | 55.00 |
| adjective | high | 2.64 | 1.33 | 63.20 |
| adjective | hot | 2.00 | 1.36 | 56.70 |
| adjective | hungry | 1.29 | 0.77 | 43.64 |
| adjective | hurt | 0.90 | 1.56 | 39.50 |
| adjective | last | 0.62 | 1.54 | 34.45 |
| adjective | little | 1.15 | 1.67 | 35.00 |
| adjective | long | 0.09 | -1.25 | 42.13 |
| adjective | loud | 1.20 | 2.77 | 35.70 |
| adjective | mad | 0.73 | 0.70 | 52.71 |
| adjective | naughty | 0.40 | 0.86 | 27.30 |
| adjective | new | -0.29 | 0.27 | 25.67 |
| adjective | nice | 0.81 | 1.08 | 36.36 |
| adjective | noisy | 1.40 | 1.00 | 37.13 |
| adjective | old | 1.27 | 1.08 | 27.09 |
| adjective | other | -0.08 | -0.50 | 27.60 |
| adjective | poor | -1.20 | 0.60 | 28.53 |
| adjective | pretty | 2.00 | 1.64 | 46.33 |
| adjective | quiet | 1.00 | -1.64 | 45.00 |
| adjective | red | 1.83 | 1.18 | 42.10 |
| adjective | sad | 1.77 | 1.31 | 46.81 |
| adjective | same | 0.00 | 0.91 | 33.40 |
| adjective | scared | 1.38 | 1.09 | 37.23 |
| adjective | sick | 2.00 | 3.50 | 46.20 |
| adjective | sleepy | 1.42 | 1.14 | 55.00 |
| adjective | slow | 1.64 | 2.53 | 34.74 |
| adjective | soft | 2.33 | 2.67 | 41.17 |
| adjective | sticky | 2.93 | 1.63 | 47.20 |
| adjective | stuck | 1.58 | 2.00 | 44.00 |
| adjective | thirsty | 1.76 | 1.00 | 59.09 |
| adjective | tiny | 3.36 | 2.55 | 48.00 |
| adjective | tired | 0.17 | 1.60 | 39.92 |
| adjective | wet | 2.25 | 0.60 | 37.79 |
| adjective | white | 0.50 | 0.57 | 33.90 |
| adjective | windy | 3.40 | 0.86 | 53.21 |
| adjective | yellow | 0.50 | 0.20 | 31.06 |
| adjective | yucky | 3.46 | 2.20 | 74.67 |
| interjection | bye | 1.58 | 0.40 | 63.86 |
| interjection | hello | 2.10 | 1.64 | 69.90 |
| interjection | hi | 0.80 | 2.70 | 65.06 |
| interjection | no | 2.81 | 3.60 | 74.67 |
| interjection | thanks | 1.42 | 1.33 | 44.25 |
| interjection | yes | 2.25 | 0.73 | 60.69 |
| function word | a | 0.46 | 1.79 | 39.36 |
| function word | about | -0.10 | -0.71 | 28.31 |
| function word | above | 1.06 | 1.13 | 38.20 |
| function word | after | 0.75 | 0.10 | 15.40 |
| function word | all | -0.10 | 1.07 | 34.21 |
| function word | am | 0.58 | -0.50 | 30.50 |
| function word | an | -0.64 | -0.30 | 16.50 |
| function word | and | 0.56 | 0.17 | 15.70 |
| function word | another | 0.12 | 0.06 | 26.30 |
| function word | any | -0.31 | -0.64 | 22.08 |
| function word | are | -0.90 | -1.25 | 18.58 |
| function word | around | 0.60 | 2.07 | 35.00 |
| function word | at | -0.17 | 0.33 | 28.35 |
| function word | away | 0.75 | 0.38 | 36.14 |
| function word | be | 0.38 | -0.46 | 30.86 |
| function word | because | 0.21 | 0.00 | 14.92 |
| function word | before | -0.50 | 1.31 | 19.50 |
| function word | behind | 1.55 | 1.15 | 36.92 |
| function word | but | 0.00 | -1.00 | 20.18 |
| function word | by | 0.27 | -0.70 | 12.50 |
| function word | could | -1.45 | 0.27 | 19.58 |
| function word | did | 0.47 | -0.15 | 37.59 |
| function word | do | 0.85 | -0.20 | 35.38 |
| function word | does | -0.60 | 0.00 | 21.20 |
| function word | down | 1.91 | NA | NA |
| function word | each | 0.67 | 0.44 | 21.20 |
| function word | every | 0.25 | 0.85 | 26.71 |
| function word | for | -1.40 | -0.09 | 18.00 |
| function word | gonna | -0.40 | 0.64 | 17.73 |
| function word | gotta | 0.00 | -0.10 | 11.69 |
| function word | he | 1.06 | 1.30 | 37.73 |
| function word | her | 0.70 | -0.50 | 53.50 |
| function word | here | -0.20 | 0.10 | 43.55 |
| function word | hers | 1.21 | 0.09 | 36.63 |
| function word | him | 0.58 | 0.60 | 49.80 |
| function word | his | 0.59 | 0.90 | 45.70 |
| function word | how | 0.46 | 0.55 | 36.00 |
| function word | I | 3.18 | 1.43 | 68.77 |
| function word | if | 0.64 | 0.77 | 28.00 |
| function word | in | 1.46 | 1.17 | 46.18 |
| function word | inside | 0.90 | 0.70 | 39.00 |
| function word | into | -0.82 | 1.21 | 32.31 |
| function word | is | -0.14 | 0.38 | 30.75 |
| function word | it | 1.00 | 1.45 | 33.07 |
| function word | later | -0.30 | 0.08 | 44.12 |
| function word | lemme | -0.90 | -1.29 | 13.14 |
| function word | me | 0.60 | 1.83 | 64.59 |
| function word | mine | 1.50 | 0.91 | 20.23 |
| function word | more | 1.13 | 1.00 | 50.74 |
| function word | much | 0.42 | 0.70 | 38.14 |
| function word | my | 1.50 | 2.60 | 40.70 |
| function word | none | 0.83 | 0.50 | 43.00 |
| function word | not | 1.38 | 0.90 | 45.58 |
| function word | now | 1.46 | -0.08 | 27.40 |
| function word | of | 0.23 | -0.36 | 26.55 |
| function word | off | 1.90 | 1.45 | 41.58 |
| function word | on | 0.92 | 1.79 | 44.73 |
| function word | our | -0.91 | 0.17 | 32.58 |
| function word | out | 0.92 | 2.67 | 42.77 |
| function word | over | 0.80 | 1.17 | 28.23 |
| function word | she | 0.71 | 1.08 | 52.40 |
| function word | so | 0.91 | 0.00 | 16.45 |
| function word | some | 0.50 | 0.20 | 30.50 |
| function word | that | -0.06 | 0.40 | 23.43 |
| function word | the | 0.43 | 1.00 | 12.09 |
| function word | their | -1.00 | -0.21 | 25.91 |
| function word | them | -0.69 | 0.25 | 43.71 |
| function word | then | 0.19 | 0.20 | 39.70 |
| function word | there | 0.46 | -0.08 | 28.12 |
| function word | these | -0.21 | -0.73 | 25.17 |
| function word | they | 0.45 | -0.73 | 47.82 |
| function word | this | 0.13 | 0.63 | 19.90 |
| function word | those | 0.27 | 0.60 | 40.65 |
| function word | to | -0.42 | 0.55 | 26.36 |
| function word | too | 1.00 | 0.53 | 24.88 |
| function word | try | -0.29 | -0.42 | 31.20 |
| function word | under | 1.18 | 1.47 | 32.20 |
| function word | up | 1.33 | 1.46 | 61.83 |
| function word | us | -0.77 | 0.79 | 51.60 |
| function word | wanna | 0.33 | 1.15 | 21.33 |
| function word | was | -0.83 | 0.55 | 23.65 |
| function word | we | 1.43 | 0.36 | 45.27 |
| function word | were | 0.60 | -0.67 | 28.30 |
| function word | what | 0.14 | -0.07 | 25.10 |
| function word | when | -1.13 | -0.62 | 18.88 |
| function word | where | -0.82 | -0.36 | 57.50 |
| function word | which | 0.09 | 0.09 | 8.91 |
| function word | who | 1.38 | 0.45 | 52.36 |
| function word | why | 0.77 | 0.20 | 36.93 |
| function word | will | 0.21 | 1.20 | 30.90 |
| function word | with | 1.17 | 0.00 | 29.70 |
| function word | would | -2.10 | -1.73 | 24.79 |
| function word | you | -0.40 | 0.18 | 56.36 |
| function word | your | 0.00 | 0.65 | 51.00 |
| function word | yourself | 0.33 | 0.42 | 34.50 |
| noun | alligator | 1.09 | 0.47 | 21.90 |
| noun | animal | -0.93 | 0.45 | 40.00 |
| noun | ankle | 0.47 | 0.09 | 29.75 |
| noun | ant | 0.60 | 1.15 | 29.30 |
| noun | apple | 0.08 | 0.69 | 28.20 |
| noun | arm | 0.20 | 0.69 | 41.67 |
| noun | aunt | 0.20 | -1.09 | 19.90 |
| noun | baby | 2.23 | 1.33 | 60.06 |
| noun | back | 0.40 | 1.00 | 58.47 |
| noun | ball | 0.75 | 1.00 | 57.70 |
| noun | balloon | 1.71 | 0.77 | 41.80 |
| noun | banana | 1.00 | 0.08 | 30.08 |
| noun | basement | 0.82 | 1.27 | 27.82 |
| noun | basket | -0.17 | 1.00 | 27.00 |
| noun | bat | 0.15 | 0.14 | 28.36 |
| noun | bath | 0.30 | 0.55 | 19.40 |
| noun | beach | -0.21 | 1.00 | 34.60 |
| noun | beads | 1.45 | 1.00 | 41.36 |
| noun | beans | 0.38 | 0.46 | 42.79 |
| noun | bear | -0.25 | 0.86 | 30.31 |
| noun | bed | 0.42 | 0.46 | 45.82 |
| noun | bee | 1.55 | 1.91 | 50.64 |
| noun | belt | 1.20 | 0.90 | 34.37 |
| noun | bench | -0.92 | 0.69 | 32.00 |
| noun | bib | 0.08 | 1.14 | 25.38 |
| noun | bird | 0.73 | 1.00 | 35.71 |
| noun | blanket | 0.12 | 0.30 | 26.27 |
| noun | block | 2.43 | 1.40 | 32.30 |
| noun | boat | 1.14 | 1.27 | 51.89 |
| noun | book | 0.09 | 0.27 | 66.55 |
| noun | boots | 0.40 | 0.71 | 43.45 |
| noun | bottle | 0.50 | 1.27 | 36.12 |
| noun | bowl | 1.50 | 1.50 | 43.00 |
| noun | box | 0.56 | 0.62 | 43.75 |
| noun | boy | 1.70 | 1.23 | 52.54 |
| noun | bread | 0.33 | 0.50 | 37.45 |
| noun | breakfast | 0.92 | 1.25 | 11.92 |
| noun | broom | 0.57 | 0.70 | 36.13 |
| noun | brother | 0.40 | 1.17 | 29.21 |
| noun | brush | 1.73 | 2.00 | 35.67 |
| noun | bubbles | 2.50 | 2.40 | 66.00 |
| noun | bucket | -0.53 | 0.30 | 42.50 |
| noun | bug | 1.90 | 2.33 | 42.18 |
| noun | bunny | 0.40 | 1.80 | 35.00 |
| noun | bus | -0.93 | -0.36 | 26.60 |
| noun | butter | 0.55 | 0.58 | 23.73 |
| noun | buttocks | 0.25 | 1.64 | 35.80 |
| noun | button | 0.50 | -0.06 | 44.67 |
| noun | cake | 0.46 | -0.17 | 52.50 |
| noun | camera | 1.33 | -0.42 | 38.36 |
| noun | camping | 0.64 | 0.90 | 20.70 |
| noun | candy | 1.12 | 0.53 | 35.00 |
| noun | car | 0.47 | 0.75 | 35.30 |
| noun | carrots | 0.50 | 0.27 | 43.00 |
| noun | cat | 0.54 | 1.13 | 46.50 |
| noun | cereal | 0.21 | -1.07 | 28.06 |
| noun | chair | 0.06 | 1.70 | 34.18 |
| noun | chalk | 0.64 | 1.00 | 37.21 |
| noun | cheek | 1.30 | 0.17 | 23.69 |
| noun | cheerios | 1.92 | 1.10 | 32.80 |
| noun | cheese | -0.36 | -0.54 | 38.83 |
| noun | child | 0.93 | 0.50 | 32.40 |
| noun | chin | 1.33 | 0.91 | 39.53 |
| noun | chocolate | 0.55 | 0.62 | 42.17 |
| noun | church | 0.08 | 0.50 | 15.91 |
| noun | circus | 0.60 | 0.09 | 29.75 |
| noun | clock | 0.61 | 1.90 | 31.75 |
| noun | closet | 1.44 | -0.14 | 29.77 |
| noun | cloud | 1.00 | -0.75 | 43.42 |
| noun | clown | 0.15 | -1.25 | 44.86 |
| noun | coat | -0.80 | -1.15 | 31.20 |
| noun | coffee | 1.30 | 0.13 | 28.06 |
| noun | coke | 1.00 | 0.25 | 23.50 |
| noun | comb | -0.07 | 0.64 | 17.91 |
| noun | cookie | 0.45 | 0.50 | 28.00 |
| noun | corn | 0.31 | -0.36 | 23.62 |
| noun | couch | -0.17 | 0.00 | 28.50 |
| noun | country | -0.53 | 0.14 | 33.00 |
| noun | cow | 1.45 | 0.50 | 45.77 |
| noun | cracker | 0.58 | 0.91 | 28.11 |
| noun | crayon | 0.82 | 0.42 | 14.50 |
| noun | crib | -0.41 | 0.50 | 15.91 |
| noun | cup | 1.54 | 0.73 | 38.87 |
| noun | daddy | 1.43 | 2.10 | 47.93 |
| noun | day | -0.15 | 1.46 | 37.53 |
| noun | deer | -0.44 | -0.21 | 49.15 |
| noun | diaper | -0.27 | 1.09 | 38.91 |
| noun | dinner | 0.26 | NA | 16.10 |
| noun | dish | 0.75 | 0.50 | 26.90 |
| noun | doctor | 1.60 | 0.81 | 27.06 |
| noun | dog | 1.27 | -0.07 | 34.00 |
| noun | doll | -0.43 | 1.79 | 33.70 |
| noun | donkey | 0.84 | 0.92 | 33.25 |
| noun | donut | 0.18 | 0.25 | 29.80 |
| noun | door | 1.27 | 0.75 | 39.33 |
| noun | drawer | -0.92 | -0.23 | 32.50 |
| noun | dress | 0.00 | 1.00 | 33.00 |
| noun | dryer | 1.56 | 1.67 | 27.92 |
| noun | duck | 0.30 | 0.60 | 40.75 |
| noun | ear | 1.10 | 1.00 | 38.78 |
| noun | egg | 1.82 | 0.40 | 49.18 |
| noun | elephant | 2.14 | -0.25 | 27.30 |
| noun | eye | 1.00 | 1.08 | 34.70 |
| noun | face | 0.27 | 0.60 | 26.40 |
| noun | farm | -0.11 | 0.73 | 32.23 |
| noun | feet | 0.36 | 0.40 | 43.06 |
| noun | finger | 0.18 | 0.43 | 62.36 |
| noun | flag | -0.79 | 0.33 | 34.91 |
| noun | flower | -0.08 | 0.91 | 40.30 |
| noun | food | 1.07 | 0.36 | 45.15 |
| noun | fork | 0.82 | 1.07 | 52.33 |
| noun | friend | 0.82 | 0.69 | 63.20 |
| noun | frog | 2.18 | 0.45 | 36.09 |
| noun | game | 0.07 | 0.82 | 26.63 |
| noun | garage | 0.13 | 0.00 | 19.50 |
| noun | garbage | 0.33 | 1.83 | 25.00 |
| noun | garden | -0.19 | 0.73 | 31.06 |
| noun | giraffe | -1.19 | 0.13 | 25.58 |
| noun | girl | 0.64 | -0.70 | 53.67 |
| noun | glass | 0.80 | 1.17 | 30.60 |
| noun | glasses | 0.42 | 0.20 | 48.86 |
| noun | gloves | -1.10 | 0.00 | 32.45 |
| noun | glue | 0.60 | 1.07 | 23.50 |
| noun | goose | 0.23 | -0.25 | 24.00 |
| noun | grapes | -0.81 | 0.00 | 46.53 |
| noun | grass | 1.10 | 0.92 | 48.68 |
| noun | gum | 1.60 | 1.00 | 43.08 |
| noun | hair | -0.42 | 0.43 | 38.50 |
| noun | hamburger | -0.36 | -0.38 | 20.08 |
| noun | hammer | 1.38 | 1.07 | 36.64 |
| noun | hand | 0.39 | 0.18 | 33.50 |
| noun | hat | 1.46 | 0.82 | 38.80 |
| noun | head | 0.69 | -0.40 | 54.88 |
| noun | hen | 0.45 | 0.45 | 32.20 |
| noun | home | 2.00 | 1.83 | 46.13 |
| noun | horse | -0.36 | 0.80 | 36.55 |
| noun | hose | 0.64 | 0.27 | 30.54 |
| noun | house | 0.45 | 0.62 | 34.00 |
| noun | ice | 0.64 | 1.08 | 42.64 |
| noun | jacket | 0.50 | -0.33 | 22.70 |
| noun | jar | 0.46 | 0.35 | 36.84 |
| noun | jeans | 0.00 | 0.17 | 30.17 |
| noun | jello | 2.25 | 1.70 | 31.20 |
| noun | jelly | 1.60 | 1.38 | 20.42 |
| noun | juice | 0.62 | 1.27 | 38.73 |
| noun | keys | 0.94 | 0.73 | 47.13 |
| noun | kitchen | -0.06 | 0.73 | 38.89 |
| noun | kitty | 0.00 | 1.64 | 53.94 |
| noun | kleenex | 2.90 | -2.18 | 23.91 |
| noun | knee | 0.13 | -0.15 | 40.70 |
| noun | knife | 0.52 | -0.90 | 41.42 |
| noun | ladder | 1.38 | 0.77 | 21.40 |
| noun | lady | -0.08 | 1.08 | 43.60 |
| noun | lamb | 0.42 | 0.27 | 25.00 |
| noun | lamp | 0.07 | 0.31 | 26.00 |
| noun | leg | 0.32 | -0.19 | 40.95 |
| noun | light | 0.90 | 1.90 | 56.78 |
| noun | lion | -0.35 | 0.00 | 42.21 |
| noun | lips | 2.31 | 1.24 | 39.23 |
| noun | lollipop | 1.33 | -0.62 | 24.17 |
| noun | lunch | 0.50 | 1.50 | 29.00 |
| noun | man | 0.65 | 1.00 | 41.00 |
| noun | meat | 0.20 | 0.27 | 48.82 |
| noun | medicine | 1.27 | 1.67 | 27.28 |
| noun | melon | -0.21 | 0.00 | 34.86 |
| noun | milk | 0.73 | -0.08 | 39.64 |
| noun | mittens | 0.00 | 0.19 | 44.38 |
| noun | mommy | 1.40 | 1.46 | 65.73 |
| noun | money | -0.60 | 1.00 | 36.20 |
| noun | monkey | 0.29 | 1.30 | 21.73 |
| noun | moon | 0.10 | 0.60 | 34.57 |
| noun | moose | 0.30 | -0.18 | 8.64 |
| noun | mop | 1.81 | 0.29 | 52.60 |
| noun | morning | -1.80 | -0.35 | 41.31 |
| noun | mouse | 0.00 | 0.55 | 30.11 |
| noun | mouth | 1.33 | 1.56 | 42.38 |
| noun | movie | 0.29 | 0.00 | 30.20 |
| noun | muffin | 1.08 | 1.08 | 31.41 |
| noun | nail | 0.18 | 0.91 | 26.50 |
| noun | napkin | -0.55 | 0.38 | 30.54 |
| noun | night | 1.09 | -0.50 | 42.70 |
| noun | noodles | 1.71 | 1.75 | 34.45 |
| noun | nose | 0.25 | 0.25 | 48.89 |
| noun | nurse | 1.00 | -0.10 | 24.90 |
| noun | nuts | 0.55 | 0.40 | 44.55 |
| noun | oven | 0.13 | 1.14 | 33.59 |
| noun | owie | 2.54 | 2.35 | 58.57 |
| noun | owl | 0.09 | 0.33 | 33.69 |
| noun | pajamas | -0.59 | -1.33 | 18.72 |
| noun | pants | -0.15 | -0.54 | 36.40 |
| noun | paper | 0.42 | 0.64 | 30.86 |
| noun | park | 0.38 | -0.18 | 17.08 |
| noun | party | 1.92 | -0.10 | 52.00 |
| noun | peas | 0.45 | 0.62 | 21.27 |
| noun | pen | 0.69 | -0.20 | 35.90 |
| noun | pencil | 0.63 | -0.90 | 27.90 |
| noun | penguin | -1.58 | -0.50 | 21.91 |
| noun | penis | -0.20 | 0.64 | 36.18 |
| noun | penny | -0.20 | -0.13 | 31.13 |
| noun | people | 0.60 | 0.92 | 50.12 |
| noun | person | -0.20 | 1.17 | 35.30 |
| noun | pickle | 1.55 | 1.00 | 36.58 |
| noun | picnic | -0.54 | -0.17 | 17.53 |
| noun | picture | -0.07 | -0.18 | 36.28 |
| noun | pig | 0.50 | 1.08 | 50.10 |
| noun | pillow | 0.47 | 0.20 | 52.47 |
| noun | pizza | 0.70 | 0.75 | 24.17 |
| noun | plant | -0.20 | 0.18 | 34.10 |
| noun | plate | 0.93 | 1.00 | 49.36 |
| noun | police | 0.06 | -0.18 | 37.38 |
| noun | pony | -0.07 | -0.45 | 38.38 |
| noun | pool | 0.30 | 0.82 | 36.45 |
| noun | popsicle | 0.50 | -0.17 | 32.00 |
| noun | porch | -0.60 | -0.50 | 27.44 |
| noun | potato | 0.86 | 0.18 | 33.67 |
| noun | potty | 0.70 | -0.08 | 31.75 |
| noun | present | 0.31 | -0.58 | 22.50 |
| noun | pretzel | 0.18 | 1.45 | 45.79 |
| noun | pudding | 0.30 | 1.00 | 8.64 |
| noun | pumpkin | -0.58 | 0.67 | 25.70 |
| noun | puppy | 0.67 | 2.73 | 32.43 |
| noun | purse | -1.64 | -0.80 | 29.23 |
| noun | puzzle | 1.67 | 0.50 | 26.10 |
| noun | radio | 0.58 | -0.42 | 39.92 |
| noun | rain | 0.33 | 0.64 | 50.60 |
| noun | raisin | 0.00 | -0.93 | 19.10 |
| noun | refrigerator | 0.57 | 0.70 | 29.23 |
| noun | rock | 1.27 | 1.45 | 54.67 |
| noun | roof | -0.25 | 1.09 | 25.20 |
| noun | room | 0.06 | 0.58 | 25.00 |
| noun | rooster | 0.83 | 0.55 | 36.42 |
| noun | salt | -0.09 | 0.08 | 24.50 |
| noun | sandwich | -0.13 | -0.46 | 32.85 |
| noun | sauce | 1.17 | 1.63 | 32.14 |
| noun | scarf | 0.42 | 0.08 | 25.67 |
| noun | school | -0.21 | 0.23 | 45.10 |
| noun | scissors | 1.75 | 0.83 | 35.40 |
| noun | sheep | 1.00 | 0.60 | 39.88 |
| noun | shirt | -0.38 | -0.70 | 33.38 |
| noun | shoe | -0.31 | 0.92 | 44.73 |
| noun | shopping | 1.41 | 0.38 | 42.54 |
| noun | shorts | 0.56 | 0.77 | 25.40 |
| noun | shoulder | -0.50 | 0.31 | 30.94 |
| noun | shovel | 1.20 | 1.27 | 47.37 |
| noun | shower | 0.94 | -0.18 | 38.82 |
| noun | sink | 0.50 | 1.10 | 29.13 |
| noun | sister | 0.53 | 0.69 | 36.00 |
| noun | sky | 0.00 | -0.50 | 37.73 |
| noun | sled | 0.60 | 1.17 | 42.12 |
| noun | slipper | 1.54 | 0.42 | 30.40 |
| noun | snack | 0.58 | 2.20 | 40.29 |
| noun | sneaker | 0.00 | -1.23 | 31.67 |
| noun | snow | 0.25 | 0.36 | 51.75 |
| noun | soap | 0.21 | 2.10 | 32.14 |
| noun | sock | 0.09 | 0.27 | 33.44 |
| noun | soda | 0.71 | 0.36 | 47.33 |
| noun | sofa | -0.40 | 0.69 | 46.50 |
| noun | soup | 1.00 | 0.27 | 41.17 |
| noun | spaghetti | 1.07 | 1.73 | 23.50 |
| noun | spoon | 0.30 | 1.45 | 36.20 |
| noun | sprinkler | 1.89 | 2.17 | 31.64 |
| noun | squirrel | 0.36 | -0.13 | 25.56 |
| noun | stairs | 1.46 | -0.21 | 46.42 |
| noun | star | 1.58 | 1.36 | 55.00 |
| noun | stick | 1.93 | 0.75 | 48.91 |
| noun | stone | 1.27 | 1.17 | 40.00 |
| noun | store | -0.88 | -0.06 | 30.93 |
| noun | story | 0.45 | 1.23 | 28.30 |
| noun | stove | -0.30 | 0.18 | 26.31 |
| noun | strawberry | 0.38 | 0.67 | 26.30 |
| noun | street | -0.18 | 0.50 | 27.00 |
| noun | stroller | 0.79 | 2.18 | 43.35 |
| noun | sun | -0.09 | -0.09 | 52.28 |
| noun | sweater | 0.40 | 1.23 | 23.60 |
| noun | table | -0.13 | 0.25 | 49.59 |
| noun | tape | 0.50 | 1.31 | 31.30 |
| noun | teacher | 0.94 | 0.58 | 51.67 |
| noun | tiger | 0.70 | 1.17 | 39.08 |
| noun | tights | 1.73 | 3.30 | 43.24 |
| noun | time | -0.70 | -0.73 | 40.42 |
| noun | tissue | 1.69 | 1.77 | 30.24 |
| noun | toast | 0.55 | 0.00 | 29.11 |
| noun | today | 0.42 | 0.79 | 24.82 |
| noun | toe | 0.14 | 0.75 | 39.40 |
| noun | tomorrow | 1.70 | 0.08 | 41.57 |
| noun | tongue | -1.00 | 0.36 | 41.73 |
| noun | tonight | 0.62 | 0.42 | 37.58 |
| noun | tooth | 1.00 | 0.94 | 37.08 |
| noun | top | 1.09 | 1.60 | 45.45 |
| noun | towel | -0.24 | 0.55 | 21.07 |
| noun | toy | 0.91 | 0.20 | 30.64 |
| noun | tractor | 0.58 | 0.67 | 17.29 |
| noun | train | -0.09 | -0.19 | 35.09 |
| noun | trash | 0.70 | 1.17 | 36.00 |
| noun | tray | -0.07 | -0.50 | 32.11 |
| noun | tree | -0.92 | -0.58 | 40.55 |
| noun | truck | 0.60 | -0.09 | 28.80 |
| noun | tummy | 2.00 | 1.18 | 49.50 |
| noun | tuna | 0.15 | -0.53 | 25.73 |
| noun | turkey | 0.07 | 0.83 | 38.32 |
| noun | turtle | 0.00 | 0.18 | 24.20 |
| noun | TV | 1.09 | 1.36 | 37.82 |
| noun | uncle | 0.17 | 1.00 | 15.39 |
| noun | vacuum | 0.10 | 1.80 | 17.44 |
| noun | vagina | -0.90 | -1.27 | 22.00 |
| noun | vanilla | 0.18 | -0.67 | 18.40 |
| noun | vitamins | 0.58 | 0.25 | 39.18 |
| noun | walker | 0.80 | 1.20 | 40.90 |
| noun | wind | 1.14 | 0.10 | 55.60 |
| noun | window | -0.83 | 0.00 | 34.60 |
| noun | wolf | 1.25 | 1.31 | 47.70 |
| noun | woods | 2.33 | 1.62 | 43.00 |
| noun | yard | -0.27 | 0.27 | 16.60 |
| noun | yesterday | 0.73 | 1.20 | 21.18 |
| noun | yogurt | -0.17 | 0.17 | 28.69 |
| noun | zebra | -0.54 | -0.36 | 18.67 |
| noun | zipper | 2.92 | 3.40 | 50.67 |
| noun | zoo | 1.54 | -0.64 | 38.00 |
| onomatopoeia | baa | 2.82 | 3.07 | 44.06 |
| onomatopoeia | choo-choo | 3.00 | 3.36 | 59.06 |
| onomatopoeia | cockadoodledoo | 3.73 | 2.33 | 47.50 |
| onomatopoeia | grr | 2.00 | 4.64 | 56.07 |
| onomatopoeia | meow | 3.50 | 3.38 | 43.10 |
| onomatopoeia | moo | 3.88 | 3.50 | 63.09 |
| onomatopoeia | ouch | 3.46 | 3.33 | 48.50 |
| onomatopoeia | peekaboo | 1.80 | 2.55 | 52.80 |
| onomatopoeia | quack | 4.08 | 4.25 | 56.00 |
| onomatopoeia | shh | 3.56 | 3.00 | 59.15 |
| onomatopoeia | uh oh | 4.36 | 2.29 | 49.73 |
| onomatopoeia | vroom | 3.55 | 3.92 | 70.67 |
| onomatopoeia | woof | 3.27 | 4.07 | 75.08 |
| onomatopoeia | yum | 2.85 | 3.44 | 67.20 |
| verb | bite | 2.00 | 1.57 | 28.70 |
| verb | blow | 1.36 | 2.00 | 43.64 |
| verb | break | 2.90 | 1.80 | 35.75 |
| verb | bring | 0.13 | 0.67 | 37.50 |
| verb | build | 1.15 | 0.86 | 26.30 |
| verb | bump | 3.36 | 2.13 | 45.33 |
| verb | buy | -0.08 | -1.54 | 20.75 |
| verb | carry | 0.00 | 0.47 | 21.69 |
| verb | catch | 0.46 | 0.85 | 42.79 |
| verb | chase | 0.69 | 0.17 | 36.25 |
| verb | clap | 2.83 | 3.09 | 67.00 |
| verb | climb | 0.85 | 1.00 | 41.90 |
| verb | close | -0.13 | 1.15 | 42.00 |
| verb | cook | 0.90 | -0.33 | 33.43 |
| verb | cover | 0.43 | 1.33 | 35.58 |
| verb | cry | 0.87 | 1.83 | 45.20 |
| verb | cut | 0.71 | 2.00 | 49.60 |
| verb | dance | 0.15 | 1.30 | 38.36 |
| verb | draw | 0.20 | 1.09 | 28.30 |
| verb | drive | 0.23 | 0.31 | 37.11 |
| verb | drop | 1.07 | 1.85 | 42.54 |
| verb | dump | 2.94 | 2.40 | 50.50 |
| verb | eat | 0.65 | 1.09 | 46.70 |
| verb | fall | 1.53 | 1.46 | 49.85 |
| verb | feed | 0.80 | 1.45 | 41.10 |
| verb | find | 0.00 | 0.40 | 22.00 |
| verb | finish | 0.18 | 0.07 | 19.58 |
| verb | fit | 0.47 | 0.75 | 27.67 |
| verb | fix | 0.54 | 0.33 | 32.73 |
| verb | get | -0.58 | 1.00 | 33.93 |
| verb | give | 1.27 | 0.20 | 29.73 |
| verb | go | 1.45 | 1.62 | 71.36 |
| verb | hate | 1.44 | 1.08 | 45.92 |
| verb | have | -0.27 | -0.30 | 22.73 |
| verb | hear | 0.07 | 0.00 | 31.92 |
| verb | help | 1.53 | 1.43 | 35.60 |
| verb | hide | 1.14 | 0.63 | 40.67 |
| verb | hit | 1.00 | 2.55 | 35.90 |
| verb | hold | 0.85 | 0.27 | 38.27 |
| verb | hug | 2.00 | 1.60 | 59.27 |
| verb | hurry | 0.75 | 2.25 | 41.00 |
| verb | jump | 1.00 | 1.36 | 53.59 |
| verb | kick | 1.95 | 2.15 | 62.09 |
| verb | kiss | 1.25 | 1.67 | 45.75 |
| verb | knock | 3.06 | 3.92 | 44.07 |
| verb | lick | 1.17 | 1.62 | 45.21 |
| verb | like | -0.33 | 0.50 | 36.05 |
| verb | listen | 0.75 | 1.19 | 28.90 |
| verb | look | 1.82 | 1.40 | 38.15 |
| verb | love | 1.13 | 1.08 | 49.10 |
| verb | make | 0.64 | 0.82 | 34.87 |
| verb | nap | 1.27 | 0.40 | 51.67 |
| verb | open | 1.10 | 1.20 | 54.17 |
| verb | paint | 1.73 | 0.79 | 33.07 |
| verb | pick | 2.08 | 2.07 | 37.92 |
| verb | play | 1.08 | 0.77 | 47.64 |
| verb | please | 0.60 | 1.73 | 26.71 |
| verb | pour | 1.30 | -0.92 | 35.00 |
| verb | pretend | -1.00 | 0.15 | 22.69 |
| verb | pull | 2.08 | 1.93 | 35.90 |
| verb | push | 2.33 | 2.40 | 55.38 |
| verb | put | 1.23 | 0.30 | 33.67 |
| verb | read | 0.09 | 1.60 | 23.60 |
| verb | ride | 1.17 | 0.82 | 19.11 |
| verb | rip | 3.74 | 2.93 | 45.77 |
| verb | run | 0.91 | 0.33 | 43.20 |
| verb | say | 0.80 | 1.60 | 46.00 |
| verb | see | 1.27 | 0.50 | 46.25 |
| verb | shake | 2.62 | 0.73 | 62.80 |
| verb | share | -0.44 | -0.70 | 31.18 |
| verb | show | 0.85 | 1.25 | 27.00 |
| verb | sing | 1.70 | 1.40 | 46.23 |
| verb | sit | 0.87 | 2.08 | 57.69 |
| verb | skate | 1.42 | 1.50 | 31.91 |
| verb | sleep | 0.77 | 0.80 | 45.83 |
| verb | smile | 1.73 | 2.80 | 55.38 |
| verb | spill | 1.92 | 1.50 | 26.00 |
| verb | splash | 3.50 | 3.69 | 58.17 |
| verb | stand | 1.50 | 1.80 | 41.80 |
| verb | stay | 0.83 | 1.42 | 50.83 |
| verb | stop | 2.50 | 2.40 | 44.50 |
| verb | sweep | 3.24 | 2.43 | 23.54 |
| verb | swim | 1.00 | 0.85 | 44.77 |
| verb | take | 1.20 | 1.70 | 62.30 |
| verb | talk | 0.21 | 0.90 | 42.58 |
| verb | taste | 1.14 | 0.92 | 27.64 |
| verb | tear | 0.56 | 1.83 | 31.50 |
| verb | think | 0.36 | 0.91 | 28.20 |
| verb | throw | 0.79 | 1.40 | 35.90 |
| verb | tickle | 2.14 | 2.31 | 37.70 |
| verb | touch | 0.82 | 0.79 | 35.42 |
| verb | turn | 0.00 | 1.36 | 26.40 |
| verb | wait | -0.50 | -0.88 | 33.45 |
| verb | wake | -0.18 | 0.47 | 30.84 |
| verb | walk | 0.73 | 0.23 | 35.25 |
| verb | wash | 1.46 | 1.50 | 45.33 |
| verb | wipe | 2.00 | 1.45 | 30.50 |
| verb | wish | 2.18 | -0.60 | 33.80 |
| verb | write | 1.18 | -1.27 | 34.68 |
