# Supplementary material for: Iconicity in English and Spanish and Its Relation to Lexical Category and Age of Acquisition
Source: PLoS One. 2015 Sep 4;10(9):e0137147. doi: 10.1371/journal.pone.0137147 (PMC4560417; doi:10.1371/journal.pone.0137147)
Supplement: S2 Table — (DOCX) [file pone.0137147.s007.docx]

S2 Table: Spanish words used in Experiments 4-5.* denotes an open class adverb that was coded as an adjective for our analyses.

| **Lexical Category** | **Spanish word** | **Experiment 4 (infinitive verbs)** | **Experiment 5 (conjugated verbs)** |
| --- | --- | --- | --- |
| adjective | alto | 0.75 | 2.00 |
| adjective | amarillo | 1.00 | 0.00 |
| adjective | azul | 0.83 | 1.73 |
| adjective | bonita | 1.21 | 3.27 |
| adjective | bueno | 2.00 | 1.13 |
| adjective | caliente | 1.92 | 2.50 |
| adjective | cansado | 0.08 | 0.64 |
| adjective | chaparro | 0.20 | 0.13 |
| adjective | chico | 1.29 | -0.20 |
| adjective | chulo | 0.00 | -0.70 |
| adjective | despierto | 1.21 | 1.18 |
| adjective | diferente | 2.36 | 1.25 |
| adjective | difícil | 1.29 | -0.20 |
| adjective | duro | 1.50 | 2.88 |
| adjective | enfermo | 1.44 | 1.57 |
| adjective | enojado | 1.69 | 0.73 |
| adjective | feliz | 2.18 | 0.90 |
| adjective | feo | 1.40 | 1.42 |
| adjective | flaco | 1.55 | 1.10 |
| adjective | frío | 1.58 | 1.80 |
| adjective | fuchi | -0.46 | -0.13 |
| adjective | fuerte | 1.35 | 2.54 |
| adjective | gordo | 2.10 | 3.18 |
| adjective | grande | 2.14 | 3.50 |
| adjective | guapo | 0.29 | -0.70 |
| adjective | igual | 1.73 | 0.73 |
| adjective | largo | 1.30 | 0.44 |
| adjective | lento | 2.36 | 1.53 |
| adjective | limpio | 0.50 | 1.18 |
| adjective | linda | 1.70 | 1.90 |
| adjective | lleno | 1.90 | 0.50 |
| adjective | malo | 1.57 | 2.60 |
| adjective | mejor | 1.42 | 1.58 |
| adjective | miedo | 0.06 | 1.20 |
| adjective | mojado | 1.82 | 1.20 |
| adjective | morado | 1.33 | 1.09 |
| adjective | negro | 2.50 | 3.20 |
| adjective | nuevo | 1.43 | 2.50 |
| adjective | oscuro | 2.75 | 1.45 |
| adjective | pegajoso | 1.60 | 1.91 |
| adjective | peligroso | 0.64 | 1.77 |
| adjective | pesado | 0.00 | 1.50 |
| adjective | pobre | 2.80 | 0.60 |
| adjective | primero | 0.58 | 2.40 |
| adjective | rojo | 1.93 | 0.54 |
| adjective | roto | 0.50 | 1.40 |
| adjective | ruidoso | 1.18 | 2.10 |
| adjective | seco | 0.00 | 1.45 |
| adjective | suave | 2.92 | 3.60 |
| adjective | sucio | 1.70 | 1.87 |
| adjective | susto | 1.17 | 0.94 |
| adjective | tonto | 1.27 | 1.50 |
| adjective | tranquilo | 2.55 | 2.70 |
| adjective | travieso | 0.57 | 0.70 |
| adjective | triste | 1.75 | -0.18 |
| adjective | último | 1.00 | 0.00 |
| adjective | vacío | -0.06 | -0.45 |
| adjective | verde | 0.15 | -0.55 |
| adjective | viejo | 0.93 | 2.40 |
| adjective* | despacio | -0.33 | 0.82 |
| function word | a | -0.45 | 0.00 |
| function word | adentro | -0.60 | 1.58 |
| function word | afuera | -1.10 | 0.17 |
| function word | ahí | 1.17 | 1.09 |
| function word | ahora | 1.57 | 0.00 |
| function word | ahorita | 0.64 | 2.64 |
| function word | al rato | -0.90 | 0.50 |
| function word | allá | 0.40 | -0.30 |
| function word | allí | 0.91 | 0.67 |
| function word | antes | 0.09 | 0.08 |
| function word | aquel | 0.73 | -0.58 |
| function word | aquella | -0.93 | 0.08 |
| function word | aquellas | -0.50 | 0.20 |
| function word | aquellos | 0.00 | 0.45 |
| function word | aquí | 0.00 | 1.79 |
| function word | arriba | 0.62 | 2.10 |
| function word | así | 1.25 | 0.30 |
| function word | atrás | 0.18 | -0.20 |
| function word | bien | 1.09 | -0.36 |
| function word | cómo | 0.33 | 0.27 |
| function word | con | 0.50 | -0.20 |
| function word | cuál | 0.29 | 0.80 |
| function word | cuándo | -0.15 | 1.10 |
| function word | de | 0.38 | -0.38 |
| function word | después | -0.13 | 0.91 |
| function word | dónde | 0.64 | -0.64 |
| function word | el | 2.18 | 0.13 |
| function word | él | 0.00 | 0.83 |
| function word | ella | 0.20 | 1.64 |
| function word | ellas | 0.86 | 1.36 |
| function word | ellos | -0.25 | 1.18 |
| function word | en | 1.62 | 0.80 |
| function word | encima | 0.07 | -0.40 |
| function word | entonces | 0.60 | -0.50 |
| function word | entre | 0.59 | 2.46 |
| function word | esa | 0.50 | 0.33 |
| function word | esas | -0.27 | 1.20 |
| function word | ese | 0.00 | 1.90 |
| function word | eso | 0.80 | 0.50 |
| function word | esos | 1.82 | 0.00 |
| function word | ésta | 0.30 | 0.20 |
| function word | estar/está | 0.27 | -0.06 |
| function word | éstas | 0.92 | 0.62 |
| function word | éste | 1.05 | 1.55 |
| function word | esto | 1.08 | 0.55 |
| function word | éstos | 0.40 | 0.64 |
| function word | haber (hay)/ha | 1.17 | 1.40 |
| function word | la | 0.64 | -0.79 |
| function word | las | 0.60 | 0.25 |
| function word | le | 0.42 | 0.50 |
| function word | lejos | 1.75 | 1.09 |
| function word | les | 0.23 | -0.19 |
| function word | lo | 0.64 | -1.00 |
| function word | los | -0.67 | 0.00 |
| function word | luego | 1.35 | 0.83 |
| function word | mal | 1.07 | 0.30 |
| function word | más | 1.56 | 1.77 |
| function word | me | 0.57 | 2.70 |
| function word | mí | 0.07 | 1.45 |
| function word | mía | 2.00 | 1.63 |
| function word | mías | 2.25 | 1.40 |
| function word | mío | 2.35 | 1.90 |
| function word | míos | 1.92 | 0.67 |
| function word | mucho | -0.33 | 2.60 |
| function word | nada | -0.92 | NA |
| function word | nosotros | 1.36 | -0.64 |
| function word | nuestro | 1.36 | 0.73 |
| function word | o | 0.67 | 1.83 |
| function word | otro | 0.80 | 0.73 |
| function word | para | 0.67 | 0.67 |
| function word | pero | 0.58 | 0.67 |
| function word | poco | 1.40 | 1.40 |
| function word | poquito | -0.71 | 0.80 |
| function word | pues | -0.40 | -1.27 |
| function word | que | 2.00 | 0.10 |
| function word | qué | 1.00 | 0.50 |
| function word | quién | 0.47 | 0.20 |
| function word | se | 0.75 | 0.10 |
| function word | ser/es | 1.14 | 0.27 |
| function word | sí | 2.24 | 2.40 |
| function word | su | 0.38 | 0.75 |
| function word | suya | 1.54 | 1.00 |
| function word | suyas | 0.50 | 0.83 |
| function word | suyo | 0.40 | 0.75 |
| function word | suyos | 0.40 | -0.27 |
| function word | te | -0.14 | 0.75 |
| function word | tí | 1.00 | -0.88 |
| function word | todo | -0.17 | 2.20 |
| function word | tu | 1.50 | 2.27 |
| function word | tuya | 1.21 | 1.50 |
| function word | tuyas | 0.86 | 0.55 |
| function word | tuyo | 1.25 | 1.10 |
| function word | tuyos | 0.60 | 0.40 |
| function word | un | 1.00 | 0.80 |
| function word | una | 1.50 | 0.60 |
| function word | unas | 0.60 | 0.57 |
| function word | unos | 0.29 | 0.67 |
| function word | y | -0.50 | 1.60 |
| function word | ya | 0.00 | 0.67 |
| function word | yo | 1.70 | 0.91 |
| interjection | adiós | 2.15 | 0.73 |
| interjection | am | 0.67 | -1.44 |
| interjection | ay | 1.33 | 1.42 |
| interjection | bravo | 1.89 | 1.60 |
| interjection | hola | 3.18 | 2.00 |
| interjection | no | 3.62 | 3.60 |
| interjection | okay | 0.74 | 3.10 |
| interjection | shhh | 2.26 | 2.56 |
| noun | abeja | 0.70 | 1.19 |
| noun | abrigo | 0.60 | 1.00 |
| noun | abuela | 1.27 | -0.45 |
| noun | abuelo | 1.46 | 1.50 |
| noun | agua | 2.64 | 2.30 |
| noun | aire | 2.08 | 2.30 |
| noun | alberca | -0.20 | 0.30 |
| noun | almohada | -0.15 | 0.40 |
| noun | amiga | 1.33 | 2.00 |
| noun | amigo | 1.75 | 2.18 |
| noun | anginas | -0.25 | -0.80 |
| noun | animal | 1.00 | 1.80 |
| noun | araña | 1.70 | -0.25 |
| noun | árbol | 1.50 | 0.53 |
| noun | ardilla | -0.58 | -1.08 |
| noun | aretes | 1.90 | -0.23 |
| noun | arroz | 0.40 | 1.83 |
| noun | aspiradora | 1.20 | 0.70 |
| noun | atole | -0.82 | -1.20 |
| noun | atún | 0.62 | -0.91 |
| noun | avión | 1.73 | 1.75 |
| noun | ayer | 0.50 | 1.60 |
| noun | azúcar | 1.15 | 1.45 |
| noun | babero | 0.31 | 0.43 |
| noun | bacinica | -1.92 | -1.20 |
| noun | banana | -0.20 | 3.08 |
| noun | bandera | 1.36 | -0.08 |
| noun | baño | 0.45 | 1.27 |
| noun | barba | 1.45 | 1.30 |
| noun | barco | 1.57 | 1.71 |
| noun | basura | 1.38 | -0.60 |
| noun | bat | -0.25 | 0.80 |
| noun | bebé | 2.60 | 1.73 |
| noun | bicho | 0.42 | 0.18 |
| noun | bicicleta | 1.75 | 2.27 |
| noun | bigote | -0.27 | 0.09 |
| noun | boca | 1.50 | 0.90 |
| noun | borrego | -0.50 | -1.10 |
| noun | bosque | 0.00 | 2.00 |
| noun | botas | 1.27 | 0.10 |
| noun | botella | 0.55 | 0.44 |
| noun | botón | 1.73 | 1.45 |
| noun | brazo | 1.25 | 0.67 |
| noun | buey | 1.08 | 0.36 |
| noun | bufanda | 0.00 | 0.00 |
| noun | búho | 0.67 | -0.56 |
| noun | burbujas | 1.36 | -0.50 |
| noun | burro | 1.27 | -0.64 |
| noun | caballo | 1.09 | 0.27 |
| noun | cabeza | 1.23 | 0.36 |
| noun | cacahuete | -0.53 | -0.85 |
| noun | cachete | 2.20 | 0.73 |
| noun | café | 2.17 | 0.60 |
| noun | caja | 0.92 | 1.25 |
| noun | cajón | 0.50 | 1.10 |
| noun | calabaza | -0.69 | 1.00 |
| noun | calcetín | -0.62 | -0.08 |
| noun | calle | 0.00 | 0.27 |
| noun | calzón | 1.90 | -0.45 |
| noun | cama | 1.30 | 0.18 |
| noun | camión | -0.53 | 0.93 |
| noun | camisa | 0.57 | 1.33 |
| noun | campo | 2.10 | 1.00 |
| noun | canasta | 1.25 | 0.40 |
| noun | cara | 1.50 | 0.30 |
| noun | carne | 2.18 | 0.50 |
| noun | carreola | 0.54 | -0.20 |
| noun | carro | 2.67 | 3.10 |
| noun | casa | 1.31 | 2.90 |
| noun | cassette | 0.09 | 0.81 |
| noun | cebra | 0.30 | 1.73 |
| noun | cepillo | 1.33 | 0.50 |
| noun | cereal | 0.71 | 1.20 |
| noun | cerillos | -0.40 | -1.09 |
| noun | cerro | 0.30 | 0.33 |
| noun | champú | -0.50 | 1.09 |
| noun | chancla | 0.71 | 0.64 |
| noun | chícharo | 0.90 | 0.43 |
| noun | chichi | -2.00 | 0.60 |
| noun | chicle | 0.83 | 0.00 |
| noun | chile | 1.83 | 1.10 |
| noun | chocolate | 2.18 | 2.27 |
| noun | chupete | 2.20 | -0.27 |
| noun | chupón | 1.10 | 2.18 |
| noun | cielo | 2.55 | 1.10 |
| noun | cigarros | 0.67 | 1.30 |
| noun | cine | 2.67 | 1.85 |
| noun | circo | 2.60 | -0.20 |
| noun | clavo | -0.75 | 0.18 |
| noun | clínica | 2.60 | 2.70 |
| noun | closet | 0.43 | 2.27 |
| noun | cobija | -1.07 | 0.08 |
| noun | coche | -0.10 | 0.67 |
| noun | cochera | 0.07 | 0.54 |
| noun | cocina | 1.07 | NA |
| noun | cocodrilo | 2.00 | 2.40 |
| noun | coladera | -0.30 | 0.30 |
| noun | collar | 0.83 | 2.09 |
| noun | colores | 1.91 | 3.60 |
| noun | columpio | 0.08 | 0.10 |
| noun | comida | 1.00 | 0.87 |
| noun | computadora | 0.92 | 2.50 |
| noun | conejo | -1.27 | -0.09 |
| noun | crayolas | 1.36 | 1.90 |
| noun | cubeta | 0.17 | 2.00 |
| noun | cuchara | 0.11 | -0.20 |
| noun | cuchillo | 1.23 | 0.82 |
| noun | cuna | 1.00 | 0.00 |
| noun | dedo | -0.20 | -0.20 |
| noun | día | 2.31 | 1.27 |
| noun | dientes | 0.60 | 1.60 |
| noun | dinero | 2.10 | 0.91 |
| noun | doctor | 1.92 | 1.45 |
| noun | ducha | 1.18 | -0.10 |
| noun | durazno | -0.30 | -1.94 |
| noun | ejotes | 0.69 | 0.00 |
| noun | elefante | 1.53 | 1.45 |
| noun | enfermera | 0.45 | 0.36 |
| noun | escalera | 0.20 | 0.50 |
| noun | escoba | 0.81 | 0.44 |
| noun | escuela | 2.30 | 1.80 |
| noun | espagueti | 1.29 | 1.60 |
| noun | espejo | 1.30 | 0.20 |
| noun | estrella | 1.50 | 1.90 |
| noun | estufa | -0.36 | 1.87 |
| noun | fábrica | 0.90 | 1.50 |
| noun | falda | 0.40 | 0.07 |
| noun | familia | 3.31 | 2.64 |
| noun | fiesta | 1.67 | 2.60 |
| noun | flor | 1.67 | 1.82 |
| noun | foca | 1.38 | -0.55 |
| noun | fotos | 1.29 | 2.47 |
| noun | fresa | 1.18 | 0.92 |
| noun | frijoles | -0.86 | 0.60 |
| noun | fuego | 1.58 | 2.70 |
| noun | galleta | 0.00 | 0.25 |
| noun | gallina | 0.07 | 0.47 |
| noun | ganso | 1.80 | 0.25 |
| noun | garganta | 3.00 | 1.00 |
| noun | gato | 0.75 | 0.90 |
| noun | gelatina | 0.29 | 2.10 |
| noun | globo | 2.40 | 1.80 |
| noun | gorra | 0.27 | 0.06 |
| noun | gracias | 2.20 | 2.25 |
| noun | guajolote | -0.57 | -1.80 |
| noun | guantes | 0.71 | 0.90 |
| noun | hambre | 0.38 | -0.20 |
| noun | hamburguesa | 2.90 | 2.33 |
| noun | helicóptero | 2.27 | 2.92 |
| noun | hermana | 2.00 | 0.30 |
| noun | hermano | 1.27 | 1.15 |
| noun | hielo | -0.79 | 0.64 |
| noun | hipopótamo | 1.62 | 2.09 |
| noun | hombro | 0.20 | -0.90 |
| noun | hormiga | -1.93 | -2.30 |
| noun | horno | 0.29 | 0.70 |
| noun | hospital | 1.69 | 2.30 |
| noun | hoy | 0.50 | 1.73 |
| noun | huevo | 0.64 | 1.13 |
| noun | iglesia | 0.70 | 1.55 |
| noun | jabón | -0.57 | 0.67 |
| noun | jamón | 0.90 | 0.80 |
| noun | jardín | 0.71 | 1.10 |
| noun | jirafa | 0.69 | -0.30 |
| noun | jugo | -1.09 | 1.40 |
| noun | juguete | 0.27 | -0.08 |
| noun | labios | 1.38 | 1.31 |
| noun | lámpara | 1.20 | 2.64 |
| noun | lápiz | 0.00 | 0.00 |
| noun | lavabo | -1.25 | 1.56 |
| noun | lavadora | 1.55 | 1.40 |
| noun | leche | 1.18 | 0.70 |
| noun | leña | -0.64 | 1.58 |
| noun | lengua | 2.15 | 1.73 |
| noun | lentes | 0.92 | 0.27 |
| noun | león | 1.00 | 1.44 |
| noun | librero | 0.20 | 0.50 |
| noun | libro | 0.70 | 0.00 |
| noun | licuado | 2.40 | 1.92 |
| noun | limonada | 2.20 | 2.08 |
| noun | llave | 0.40 | -0.53 |
| noun | lluvia | 1.27 | 0.50 |
| noun | lobo | 0.67 | 0.38 |
| noun | luna | 1.77 | 2.00 |
| noun | luz | 1.50 | 2.08 |
| noun | maceta | -1.00 | -0.20 |
| noun | madrina | 0.40 | 0.90 |
| noun | maestra | 0.62 | 0.75 |
| noun | mamá | 3.46 | 1.92 |
| noun | mamila | -0.29 | 1.45 |
| noun | mañana | 1.79 | 0.00 |
| noun | manguera | 1.27 | 0.64 |
| noun | maní | 0.57 | -0.36 |
| noun | mano | -0.13 | 1.27 |
| noun | mantequilla | -0.31 | 0.55 |
| noun | manzana | 0.53 | -0.73 |
| noun | mariposa | 1.86 | 1.20 |
| noun | martillo | -0.07 | 1.64 |
| noun | medias | -0.92 | 0.55 |
| noun | medicina | 0.85 | 3.09 |
| noun | melón | 1.33 | 2.40 |
| noun | mercado | 1.91 | 0.67 |
| noun | mermelada | 1.60 | 2.09 |
| noun | mesa | 1.00 | 1.17 |
| noun | mono | 0.50 | 0.38 |
| noun | mosca | 0.23 | -0.46 |
| noun | mosco | 0.82 | 0.08 |
| noun | moto | 0.30 | 2.64 |
| noun | mueble | 1.08 | -1.25 |
| noun | muñeca | 1.09 | -0.21 |
| noun | nalgas | 1.20 | 0.09 |
| noun | nana | -0.18 | 0.83 |
| noun | naranja | 1.67 | 1.20 |
| noun | nariz | 1.43 | 1.91 |
| noun | niña | 2.27 | 1.31 |
| noun | niño | 2.60 | 1.10 |
| noun | noche | 1.71 | 2.09 |
| noun | nube | 1.82 | 1.60 |
| noun | oficina | 1.79 | 1.50 |
| noun | ojitos | 1.18 | 1.50 |
| noun | ojos | 0.09 | 1.18 |
| noun | olla | -1.29 | 0.00 |
| noun | ombligo | -0.14 | -1.54 |
| noun | oreja | 1.92 | 0.33 |
| noun | osito | 2.00 | 0.60 |
| noun | oso | 0.40 | -0.31 |
| noun | padrino | 1.62 | 0.69 |
| noun | pájaro | 0.00 | 0.40 |
| noun | pala | 0.10 | 0.13 |
| noun | paleta | -0.20 | 1.10 |
| noun | palo | -0.21 | 1.08 |
| noun | palomitas | 1.45 | 0.27 |
| noun | pan | 1.60 | 0.67 |
| noun | pan dulce | 2.30 | 2.10 |
| noun | pañal | 0.91 | -0.71 |
| noun | pantalón | 0.00 | 0.00 |
| noun | pañuelo | -1.40 | -0.75 |
| noun | panza | 0.30 | -0.30 |
| noun | papá | 1.80 | 1.70 |
| noun | papas | 1.47 | 0.50 |
| noun | papel | 2.56 | 1.00 |
| noun | papitas | 2.40 | 1.00 |
| noun | parque | 0.25 | 2.75 |
| noun | pasas | 0.14 | 0.30 |
| noun | pastel | 1.54 | 1.27 |
| noun | pasto | 0.00 | -0.60 |
| noun | patines | -0.09 | -0.10 |
| noun | pato | 1.64 | -0.55 |
| noun | payaso | 1.00 | 0.55 |
| noun | peine | 1.25 | -0.70 |
| noun | pelo | 0.45 | 1.20 |
| noun | pelota | 0.18 | 1.06 |
| noun | pene | 0.92 | -0.75 |
| noun | periódico | 2.21 | 1.00 |
| noun | perro | 1.57 | -0.18 |
| noun | persona | 1.55 | 2.91 |
| noun | piedra | -0.40 | 1.20 |
| noun | piernas | -0.08 | -0.20 |
| noun | pies | -0.92 | 1.18 |
| noun | pijama | 2.21 | 0.40 |
| noun | pingüino | -0.14 | 1.38 |
| noun | piscina | -1.27 | -0.64 |
| noun | plancha | 1.36 | -0.18 |
| noun | planta | 1.45 | 1.08 |
| noun | plastilina | 1.00 | 1.10 |
| noun | plátano | 0.82 | 1.17 |
| noun | plato | 1.00 | 1.30 |
| noun | playa | 0.42 | -1.00 |
| noun | playera | -0.50 | -0.46 |
| noun | policía | 1.33 | 1.70 |
| noun | pollito | 1.73 | 0.90 |
| noun | pollo | 1.64 | 1.17 |
| noun | prima | 0.93 | 0.60 |
| noun | primo | -0.14 | -0.20 |
| noun | puerco | 0.85 | 1.30 |
| noun | puerta | -0.08 | 1.33 |
| noun | quesadilla | 2.73 | 1.30 |
| noun | queso | 1.33 | 1.90 |
| noun | radio | 1.20 | 2.55 |
| noun | rana | 1.30 | 1.45 |
| noun | rancho | 2.45 | 2.55 |
| noun | ratón | 1.00 | 1.54 |
| noun | recámara | -0.43 | -0.08 |
| noun | refresco | 1.71 | 1.36 |
| noun | refrigerador | 2.46 | 0.88 |
| noun | regadera | -0.75 | -0.64 |
| noun | reja | -0.45 | 0.50 |
| noun | reloj | -0.90 | -0.85 |
| noun | resbaladilla | 1.10 | -0.75 |
| noun | río | 1.78 | -1.00 |
| noun | rodilla | 0.00 | -0.42 |
| noun | ropa | 0.86 | 0.70 |
| noun | ropero | 1.31 | 0.47 |
| noun | sal | 1.31 | 1.18 |
| noun | sala | 0.15 | 0.60 |
| noun | salchicha | 1.00 | -0.30 |
| noun | salsa | 2.55 | 2.18 |
| noun | sandía | -1.07 | -1.20 |
| noun | secadora | 1.36 | 0.80 |
| noun | señor | 2.57 | 2.10 |
| noun | señora | 2.44 | 0.91 |
| noun | servilleta | 1.00 | 0.40 |
| noun | shorts | -0.40 | 1.45 |
| noun | silla | 0.83 | 0.75 |
| noun | sillón | -0.18 | 1.09 |
| noun | soda | 1.38 | 2.92 |
| noun | sofá | 1.10 | 1.42 |
| noun | sol | 1.70 | 1.80 |
| noun | sombrero | 1.57 | 1.87 |
| noun | sopa | 1.55 | 1.33 |
| noun | suéter | -0.08 | 0.91 |
| noun | taco | 1.36 | 0.90 |
| noun | tambor | 1.64 | 1.60 |
| noun | tanque | 1.40 | 1.50 |
| noun | tapete | 0.00 | -0.27 |
| noun | taza | 0.45 | 1.00 |
| noun | té | 1.20 | 0.18 |
| noun | techo | 1.07 | 0.70 |
| noun | teléfono | 1.88 | 2.90 |
| noun | televisión | 3.00 | 3.40 |
| noun | templo | 1.57 | 3.64 |
| noun | tenedor | -0.27 | 0.50 |
| noun | tía | 0.90 | 0.10 |
| noun | tienda | 0.47 | 0.60 |
| noun | tierra | 1.33 | 1.50 |
| noun | tigre | 1.30 | 1.77 |
| noun | tijeras | -0.73 | 0.10 |
| noun | timbre | 1.57 | 2.07 |
| noun | tina | -1.15 | 0.06 |
| noun | tío | 0.71 | -0.17 |
| noun | toalla | -0.73 | 0.85 |
| noun | torta | 1.77 | 0.27 |
| noun | tortilla | -0.73 | 2.09 |
| noun | tortillitas | 2.82 | 2.36 |
| noun | tortuga | 1.09 | 1.63 |
| noun | tractor | 2.00 | 2.70 |
| noun | trapo | 2.43 | 0.33 |
| noun | tren | 2.24 | 1.92 |
| noun | trineo | 1.00 | -1.58 |
| noun | troca | 1.73 | 1.80 |
| noun | uvas | 1.80 | 0.20 |
| noun | vaca | -0.40 | 0.18 |
| noun | vagina | 0.10 | 0.75 |
| noun | vainilla | 0.17 | 1.55 |
| noun | vasos | -2.00 | 1.54 |
| noun | vela | -1.19 | -0.40 |
| noun | venado | 0.64 | -0.60 |
| noun | ventana | 1.45 | -0.21 |
| noun | vestido | 1.10 | 1.67 |
| noun | víbora | 0.40 | 0.90 |
| noun | viento | 0.86 | 1.50 |
| noun | vitaminas | 2.54 | 2.18 |
| noun | yoghurt | -0.10 | 0.30 |
| noun | zanahoria | -0.91 | -0.75 |
| noun | zapato | 0.23 | 0.60 |
| noun | zoológico | 1.33 | 3.60 |
| onomatopoeia | cuacuá | 3.00 | 1.58 |
| onomatopoeia | ee | -0.22 | -1.54 |
| onomatopoeia | guaguá | 1.33 | 0.27 |
| onomatopoeia | mee | 1.11 | 0.40 |
| onomatopoeia | miau | 2.84 | 0.73 |
| onomatopoeia | muu | 1.80 | -0.50 |
| onomatopoeia | pío pío | 2.63 | 2.90 |
| onomatopoeia | pipí | 3.00 | 1.60 |
| onomatopoeia | pum | 0.27 | 0.64 |
| onomatopoeia | quiquiriquí | 2.86 | 2.00 |
| onomatopoeia | tutú | 0.20 | -0.64 |
| verb | abrir/abre | 1.93 | 0.00 |
| verb | acabar/acaba | -0.23 | -0.27 |
| verb | acompañar/acompaña | 1.91 | 0.70 |
| verb | acostar/acuesta | 0.00 | 1.73 |
| verb | agarrar/agarra | 1.00 | 0.07 |
| verb | almorzar/almuerza | -0.60 | 0.50 |
| verb | amarrar/amarra | 0.47 | 2.73 |
| verb | andar/anda | -0.14 | 0.91 |
| verb | apagar/apaga | -1.00 | 0.00 |
| verb | apurar/apura | 0.36 | -0.50 |
| verb | asustar/asusta | 0.75 | 1.50 |
| verb | aventar/aventa | 0.41 | 0.55 |
| verb | ayudar/ayuda | 1.17 | 1.82 |
| verb | bailar/balla | 2.40 | 0.30 |
| verb | barrer/barre | 0.17 | 0.00 |
| verb | besar/besa | 1.18 | -0.08 |
| verb | besitos | 1.75 | 2.33 |
| verb | brincar/brinca | -0.14 | 0.80 |
| verb | buscar/busca | 1.45 | 0.67 |
| verb | caber/cabe | 0.09 | -0.91 |
| verb | caer/cae | 0.00 | 0.30 |
| verb | callar/calla | 0.90 | 1.00 |
| verb | caminar/camina | 0.60 | 0.10 |
| verb | cantar/canta | 0.92 | 0.58 |
| verb | cargar/carga | 0.17 | 0.73 |
| verb | cenar/cena | 1.33 | 0.60 |
| verb | cerrar/cerra | 0.40 | 0.85 |
| verb | cocinar | 1.62 | NA |
| verb | comer/come | 1.36 | 0.09 |
| verb | comprar/compra | 0.06 | -0.50 |
| verb | correr/corre | -0.90 | 0.80 |
| verb | cortar/corta | 2.33 | 1.31 |
| verb | cosquillitas | 1.29 | 1.18 |
| verb | dar/da | 0.33 | -0.60 |
| verb | decir/dice | 1.23 | 0.20 |
| verb | dibujar/dibuja | 0.73 | -1.25 |
| verb | doler/dole | -1.54 | -0.70 |
| verb | dormir/duerme | 2.08 | 1.20 |
| verb | empujar/empuja | 0.95 | 0.73 |
| verb | encontrar/encuentra | 0.00 | 0.20 |
| verb | enseñar/enseña | 0.20 | 0.67 |
| verb | entrar/entra | 2.21 | 1.93 |
| verb | equivocar/equivoca | 0.21 | -1.36 |
| verb | esconder/esconde | 0.55 | -0.30 |
| verb | escribir/escribe | 0.92 | 1.36 |
| verb | escuchar/escucha | 0.85 | 0.60 |
| verb | esperar/espera | 0.64 | 1.18 |
| verb | ganar/gana | 1.15 | 1.09 |
| verb | gritar/grita | 0.15 | 1.21 |
| verb | gustar/gusta | 1.09 | 1.25 |
| verb | hacer/hace | 0.10 | -0.40 |
| verb | ir/va | 1.07 | 1.70 |
| verb | jalar/jala | 0.00 | 0.40 |
| verb | jugar/juega | 1.18 | 1.30 |
| verb | juntar/junta | 0.77 | -0.13 |
| verb | lastimar/lastima | 0.00 | 2.64 |
| verb | lavar/lava | 2.30 | -0.36 |
| verb | leer/lee | 0.17 | -1.30 |
| verb | levantar/levanta | 1.30 | 0.88 |
| verb | llevar/lleva | 0.46 | 1.00 |
| verb | llorar/llora | 1.60 | 0.94 |
| verb | llover/llueve | 0.71 | -0.23 |
| verb | meter/mete | 1.36 | 0.50 |
| verb | mirar/mira | 2.09 | 2.00 |
| verb | morder/muerde | 0.58 | 0.13 |
| verb | nadar | 2.46 | NA |
| verb | oír/oye | 1.31 | 2.08 |
| verb | patear/patea | 1.40 | 0.00 |
| verb | patinar/patina | 1.50 | 0.00 |
| verb | pegar/pega | 0.50 | 0.91 |
| verb | peinar/peina | 0.20 | -0.13 |
| verb | pensar/piensa | 1.82 | 0.31 |
| verb | perder/pierde | 1.09 | -0.23 |
| verb | pintar/pinta | 1.45 | 0.58 |
| verb | platicar/platica | 0.08 | -0.40 |
| verb | poner/pone | -0.21 | -0.06 |
| verb | prender/prende | 0.43 | 0.80 |
| verb | quedar/queda | -0.47 | -0.25 |
| verb | quemar/quema | -0.29 | -0.60 |
| verb | querer/quiere | 0.29 | -0.18 |
| verb | quitar/quita | 1.46 | 1.40 |
| verb | regalar/regala | 0.79 | 0.64 |
| verb | romper/rompe | 1.25 | 1.31 |
| verb | saber/sabe | 0.10 | 1.10 |
| verb | sacar/saca | 1.00 | 0.42 |
| verb | salir/sale | 0.46 | -0.67 |
| verb | saltar/salta | 0.10 | 0.64 |
| verb | saludar/saluda | 1.83 | 0.67 |
| verb | sentar/sienta | 0.70 | 1.27 |
| verb | siesta | 0.82 | 1.25 |
| verb | soplar/sopla | 0.69 | 0.92 |
| verb | subir/sube | 1.92 | 1.73 |
| verb | tapar/tapa | -0.18 | 2.00 |
| verb | tener/tiene | -0.09 | 0.08 |
| verb | terminar/termina | 0.67 | 1.00 |
| verb | tirar/tira | 0.54 | -0.80 |
| verb | tocar/toca | 1.50 | 1.50 |
| verb | tomar/toma | -0.38 | 0.67 |
| verb | traer/trae | 0.00 | 0.42 |
| verb | vámonos | 0.62 | 0.83 |
| verb | venir/viene | 1.58 | 0.06 |
| verb | ver/ve | 0.59 | -1.00 |
